# Biomolecular Fluorescence Complementation Profiling and Artificial Intelligence Structure Prediction of the Kaposi’s Sarcoma-Associated Herpesvirus ORF18 and ORF30 Interaction

**DOI:** 10.3390/ijms23179647

**Published:** 2022-08-25

**Authors:** Yoshiko Maeda, Tadashi Watanabe, Taisuke Izumi, Kazushi Kuriyama, Shinji Ohno, Masahiro Fujimuro

**Affiliations:** 1 Department of Cell Biology, Kyoto Pharmaceutical University, Kyoto 607-8412, Japan; 2 Department of Virology, Graduate School of Medicine, University of the Ryukyus, Okinawa 903-0215, Japan; 3 Department of Biology, Saint Joseph’s University, Philadelphia, PA 19104, USA

**Keywords:** Kaposi’s sarcoma-associated virus (KSHV), viral pre-initiation complex (vPIC), bimolecular fluorescence complementation (BiFC), artificial intelligence (AI) structure prediction, AlphaFold2

## Abstract

Kaposi’s sarcoma-associated herpesvirus (KSHV) is the etiologic agent of Kaposi’s sarcoma, primary effusion lymphoma (PEL), and multicentric Castleman’s disease. During KSHV lytic infection, lytic-related genes, categorized as immediate-early, early, and late genes, are expressed in a temporal manner. The transcription of late genes requires the virus-specific pre-initiation complex (vPIC), which consists of viral transcription factors. However, the protein-protein interactions of the vPIC factors have not been completely elucidated. KSHV ORF18 is one of the vPIC factors, and its interaction with other viral proteins has not been sufficiently revealed. In order to clarify these issues, we analyzed the interaction between ORF18 and another vPIC factor, ORF30, in living cells using the bimolecular fluorescence complementation (BiFC) assay. We identified four amino-acid residues (Leu29, Glu36, His41, and Trp170) of ORF18 that were responsible for its interaction with ORF30. Pull-down assays also showed that these four residues were required for the ORF18-ORF30 interaction. The artificial intelligence (AI) system AlphaFold2 predicted that the identified four residues are localized on the surface of ORF18 and are in proximity to each other. Thus, our AI-predicted model supports the importance of the four residues for binding ORF18 to ORF30. These results indicated that wet experiments in combination with AI may enhance the structural characterization of vPIC protein-protein interactions.

## 1. Introduction

KSHV, of which DNA fragments were initially detected from a Kaposi’s sarcoma lesion from an AIDS patient in 1994, was identified as the 8th human herpesvirus [1]. Currently, KSHV is known to be the causative factor of several malignancies, including Kaposi’s sarcoma, PEL, and multicentric Castleman’s disease [2,3,4]. Recently, it has been reported that KSHV can infect nervous system cells and induce neurological disorders [5]. In addition, KSHV infection of neurons have been detected in HIV-positive patients [6] and the association of KSHV infection with neural diseases and neurological complications has been observed [7,8]. The human herpesviruses are classified into the following three subfamilies: *alphaherpesvirinae* [herpes simplex virus-1 (HSV-1), herpes simplex virus-2 (HSV-2), and varicella-zoster virus (VZV)], *betaherpesvirinae* [human cytomegalovirus (HCMV), human herpesvirus-6A (HHV-6A), human herpesvirus-6B (HHV-6B), and human herpesvirus-7 (HHV-7)], and *gammaherpesvirinae* [Epstein-Barr virus (EBV) and KSHV] [9]. These human herpesviruses have a highly conserved replication system; however, their pathological features are divergent.

The herpesviral vPIC is the essential transcriptional machinery for late gene expression and it is highly conserved between *betaherpesvirinae* and *gammaherpesvirinae*. Recently, much attention has been focused on the importance of the vPIC for viral replication or production [10]. The vPIC consists of several viral proteins that are recruited to the transcription start site (TSS) of the viral late genes, which encode viral structural proteins (e.g., envelope, capsid, and tegument proteins) that participate in viral assembly. The vPIC recognizes the TATT motif in the TSS instead of a TATA-box and initiates pre-mRNA elongation in conjunction with cellular RNA polymerase II (RNAPII). Although the host cell pre-initiation complex (which consists of various general transcription factors, GTFs) is a functional homolog of vPIC, the amino-acid sequence homology between GTF and vPIC components is quite low. Therefore, the vPIC formation machinery and the precise roles of each vPIC component have remained largely unknown. In the case of KSHV, the vPIC is thought to consist of at least six viral components: ORF18, ORF24, ORF30, ORF31, ORF34, and ORF66 [11,12,13,14,15,16]. It has been reported that the KSHV ORF24 binds to the promoters of the KSHV late genes along with cellular RNAPII [11]. Furthermore, ORF34 is thought to act as a hub for interaction with other vPIC components including ORF18, ORF24, ORF31, and ORF66 [11,12,13,14,15,16]. Several studies have determined that ORF18 interacts with ORF30, ORF31, ORF34, and ORF66 [11,12,13,14]. Namely, these previous studies indicate that ORF18 might serve as a bridge molecule between ORF30 and ORF31/ORF34/ORF66. Although ORF18 is a key element for KSHV vPIC formation, the binding partners of ORF18 have not been fully revealed. Therefore, to gain insight into the molecular interaction involved in ORF18, we applied the following two approaches: the BiFC assay and AI structure prediction. The BiFC assay employs split-fluorescent protein reconstitution to image protein-protein interactions in living cells [17]. Various methods for protein structure prediction in silico have been previously developed. However, the application of AI with the deep learning algorithm AlphaFold2 has led to a paradigm shift, and more accurate whole protein structures were obtained [18,19,20].

As mentioned above, the components of the KSHV vPIC and its functions in the transcription of late genes are now becoming clear. However, the protein-protein interactions of the KSHV vPIC factors including ORF18 have not been sufficiently revealed. In order to address these questions, we analyzed the interaction(s) of ORF18 with other vPIC factors. Initially, we analyzed the interactions between KSHV ORF18 and the other components of vPIC using a BiFC assay. Our results showed that apparent BiFC-positive signals were detected between ORF18 and ORF30. Furthermore, we identified four amino-acid residues within ORF18 that were responsible for its interaction with ORF30. Meanwhile, the AI system AlphaFold2 predicted the KSHV ORF18 structure with a high confident algorithm score. The AI-predicted ORF18 structure showed that the four identified amino-acids were exposed to the surface of ORF18 and located in proximity to each other. These four residues could be thought to support the surface structure of ORF18 required for binding to ORF30. Thus, the AI-predicted structural model would imply that these four residues of ORF18 might be involved in the interaction with ORF30.

## 2. Results

### 2.1. Optimization of the BiFC Assay to Assess vPIC Interactions

**Figure 1:**
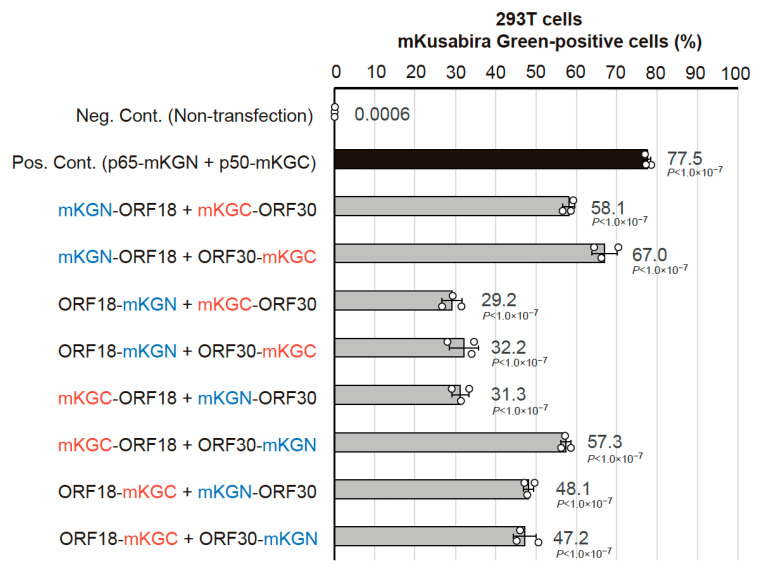
Optimization of the BiFC assay to assess the interaction between KSHV ORF18 and ORF30. In order to optimize the addition of a tag to ORF18 and ORF30, we used different types of split-mKusabira Green (mKG) tag (mKGN or mKGC) and different locations of the tag (N-terminus or C-terminus). Each indicated combination of the expression plasmids was co-transfected into 293T cells by the calcium phosphate method. The mKG-signal was assessed by flow cytometry in three independent samples, which are indicated by the white dots. We utilized non-transfected cells as a negative control (Neg. Cont.). The positive control (Pos. Cont.) was cells co-transfected with p65-mKGN (pCONT-1) and p50-mKGC (pCONT-2) expression plasmids. The average of the mKG-positive cells (%) are noted beside the bars. Each bar and error bar indicate the average and standard deviation, respectively. The statistical significance between each combination and the negative control was analyzed with one-way ANOVA followed by Tukey’s test. The *p* values are shown beside the bars.

Generally, there is a possibility that a tag fused to a protein of interest (P.O.I.) may influence its physical interactions with other proteins. Additionally, in the BiFC assay, inappropriate tag orientation increases the physical distance between the split fluoro-proteins and inhibits fluorescence reconstitution, despite interactions between P.O.I.s. Thus, in the BiFC assay, these fused tags may produce pseudo-negative signals or reduced positive signals. Therefore, to eliminate protein interaction interference by a fused tag and its orientation, all the combinations of fused tags (i.e., the type of tag and its location) were constructed as expression plasmids and analyzed for optimization of the BiFC assay. KSHV vPIC components (ORF24, ORF30, ORF31, ORF34, and ORF66) were screened for ORF18 binding with the BiFC assay. The indicated combination of expression plasmids was transfected into 293T cells, and the cells were subsequently subjected to flow cytometry. Harvested cells were gated by forward scatter (FSC) and side scatter (SSC) parameters to exclude debris, and the ratio of mKusabira-green (mKG)-positive cells were further analyzed. We detected a robust ORF18-ORF30 binding signal from all of the transfected cells. The combination and orientation of BiFC tags had various effects on the positive scores, ranging from approximately 30% to approximately 70%. Co-expression of N-terminally mKGN-tagged ORF18 (mKGN-ORF18) and C-terminally mKGC-tagged ORF30 (ORF30-mKGC) was the pair with the highest score (Figure 1). However, significant positive signals were not obtained between ORF18 and other vPIC components (Supplemental Figure S1a,b). Consequently, we further examined the binding characteristics of ORF18-ORF30.

### 2.2. Identification of the Regions of ORF18 That Interact with ORF30

**Figure 2:**
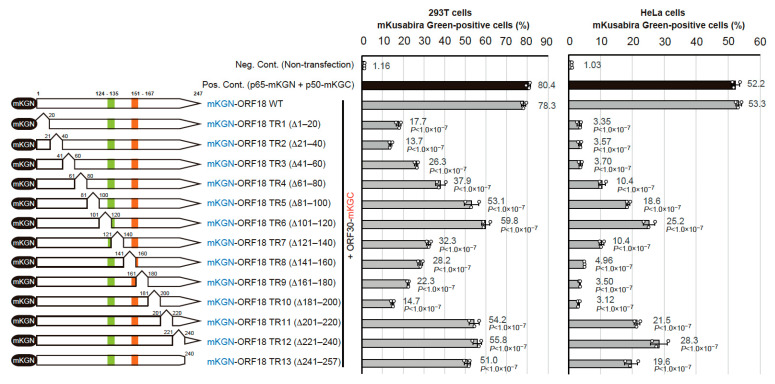
Identification of the ORF30-binding site on ORF18 using truncated mutants. The N-terminally mKGN-tagged ORF18 truncated region (TR) mutants (mKGN-ORF18 TR1 to TR13) are graphically displayed. The green block on ORF18 (amino-acid 124–136) indicates the basic region, and the orange block (amino-acid 151–169) indicates the hydrophobic region. These regions were defined by a previous report describing a murine gammaherpesvirus homolog [21]. Each mKGN-ORF18 truncated mutant expression plasmid was co-transfected with ORF30-mKGC into 293T and HeLa cells by a lipofection method and three independent samples were assessed by flow cytometry. We utilized non-transfected cells as a negative control (Neg. Cont.). The positive control (Pos. Cont.) was cells co-transfected with p65-mKGN (pCONT-1) and p50-mKGC (pCONT-2) expression plasmids. The average of the mKusabira Green-positive cells (%) are noted beside the bars. Each bar and error bar indicate the average and standard deviation, respectively. The statistical significance between each ORF18 mutant and ORF18 WT was analyzed with one-way ANOVA followed by Tukey’s test. The *p* values are also shown beside the bars. The protein expression levels of the assessed mutants are shown in Supplemental Figure S2.

In order to identify the regions of ORF18 that interact with ORF30, we constructed a total of 13 ORF18 truncated mutants. These truncations consisted of truncated region 1 (TR1) to truncated region 13 (TR13). Each truncation consisted of approximately 20 amino-acid residues that were deleted from the N-terminus to the C-terminus of ORF18. The basic region and hydrophobic region previously reported in the murine gammaherpesvirus homolog are also depicted in Figure 2 (left panel). Truncation of these regions failed to express viral capsid proteins [21]. If the truncated regions are important for the association with ORF30, the positive BiFC signal might be reduced or lost. Each mKGN-ORF18 truncated mutant was co-transfected with ORF30-mKGC into 293T cells (Figure 2; center panel) or HeLa cells (Figure 2; right panel) and analyzed. Both cell lines showed similar results, ORF18 truncated mutants scoring under 30% in 293T cells and under 5% in HeLa cells were considered binding-negative mutants. Thus, the following binding-negative mutants were observed: TR1 (∆ 1–20 amino-acid), TR2 (∆ 21–40 amino-acid), TR3 (∆ 41–60 amino-acid), TR8 (∆ 141–160 amino-acid), TR9 (∆ 161–60 amino-acid), and TR10 (∆ 141–200 amino-acid). Therefore, this data suggested that amino-acid residues 1 to 60 and amino-acid residues 141 to 200 of ORF18 are essential regions for ORF30-binding.

### 2.3. Alignment of KSHV ORF18 to Its Viral Homologs

**Figure 3:**
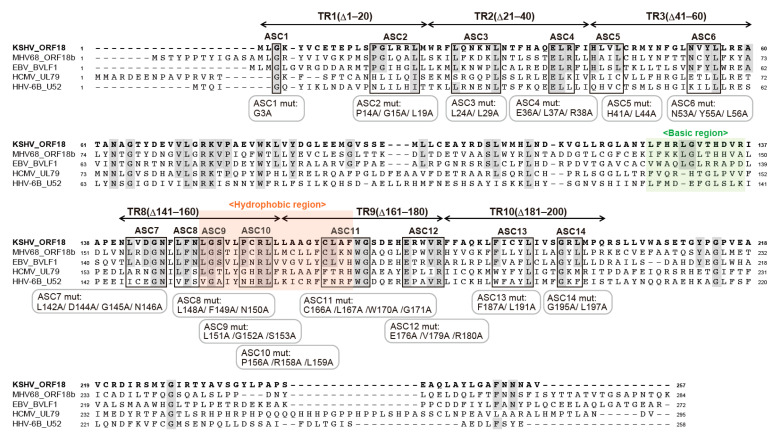
KSHV ORF18 amino-acid sequence alignment. Each herpesvirus homologous amino-acid was translated from the nucleotide sequences from the NCBI database [KSHV ORF18 (JSC-1-BAC16; Accession number GQ994935), MHV68 ORF18b (strain WUMS; NC_001826), EBV BVLF1 (strain Akata; KC207813), HCMV UL79 (strain Towne; FJ616285), and HHV-6B U52 (strain japan-a1; KY239023)]. The alignment raw data were obtained using Clustal Omega (EMBL-EBI; https://www.ebi.ac.uk/Tools/msa/clustalo/ (accessed on 14 December 2021)). The regions of TR1-3 and TR8-10 were essential for the interaction with ORF30 in the BiFC interaction assay (Figure 2). The conserved amino-acid residues between homologs, at least completely conserved in the gammaherpesvirus family (KSHV, MHV68, and EBV), are indicated with a gray background. Based on the amino-acid homology, several conserved amino-acid residues were split into blocks (ASC1-ASC14) and each conserved amino-acid was substituted with an alanine thus generating the alanine-scanning ORF18 mutants, ASC1mut–ASC14mut. These mutants contain the following substitutions: ASC1mut; ORF18 G3A, ASC2mut; ORF18 P14A/G15A/L19A, ASC3mut; ORF18 L24A/L29A, ASC4mut; ORF18 E36A/L37A/R38A, ASC5mut; ORF18 H41A/L44A, ASC6mut; ORF18 N53A/Y55A/L56A, ASC7mut; ORF18 L142A/D144A/G145A/N146A, ASC8mut; ORF18 L148A/F149A/N150A, ASC9mut; ORF18 L151A/G152A/S153A, ASC10mut; ORF18 P156A/R158A/L159A, ASC11mut; ORF18 C166A/L167A/W170A/G171A, ASC12mut; ORF18 E176A/V179A/R180A, ASC13mut; ORF18 F187A/L191A, and ASC14mut; ORF18 G195A/L197A. As depicted in Figure 2, the basic region is shown in green, and the hydrophobic region is shown in orange.

In order to survey the amino-acid residues of ORF18 which facilitate its binding to ORF30, whole amino-acid sequences of KSHV ORF18 were compared to its homologs from the betaherpesviruses (HCMV and HHV6B) as well as the gammaherpesviruses (MHV68 and EBV) (Figure 3). Conserved amino-acid sequences among all homologs or those exclusively conserved in gammaherpesviral homologs were indicated in the alignment. The regions (TR1–3 and TR8–10) identified in Figure 2 are also illustrated. Based on this information, we selected several adjacent conserved amino-acid residues in the narrowed regions and designated them as blocks. Each of these 14 blocks (ASC1–ASC14) were subjected to alanine-scanning mutagenesis, where the conserved amino-acid residues in each block were substituted with alanine residues, which is denoted as ASC1mut–ASC14mut underneath the alignment. These mutants were used in the next experiments.

### 2.4. Narrowing the ORF18 Interaction Blocks to Single Amino-Acid Residues

**Figure 4:**
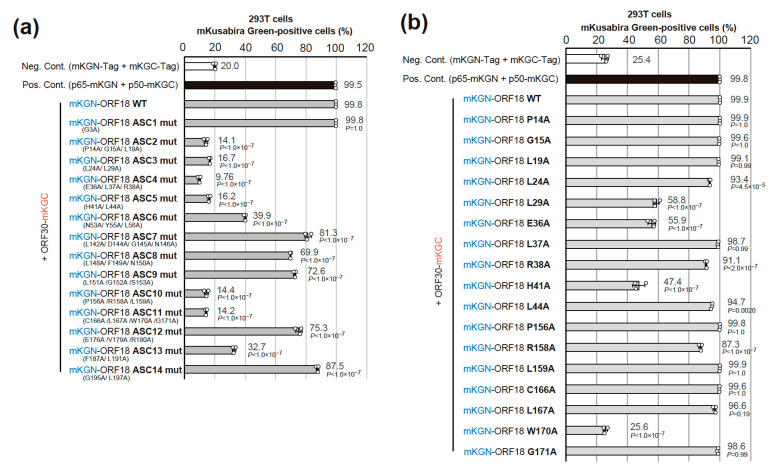
Identification of the ORF30-binding site on ORF18 using block alanine-scanning and single alanine-scanning mutants. (**a**) The indicated mKGN-ORF18 block alanine-scanning mutant (mKGN-ORF18 ASC1mut to ASC14mut) expression plasmid was co-transfected with ORF30-mKGC and mCherry, which was used as a transfection control. (**b**) The indicated mKGN-ORF18 single alanine-scanning mutant expression plasmid was co-transfected with ORF30-mKGC and mCherry, which was used as a transfection control. For both (**a**) and (**b**), 293T cells were transfected by a lipofection method and three independent samples were assessed by flow cytometry. The individual mutations that comprise the block alanine-scanning mutation are shown under the mKGN-ORF18 expression plasmid name. The negative control (Neg. Cont.) was a combination of tag-only expression plasmids (mKGN and mKGC), and the positive control (Pos. Cont.) was a combination of p65-mKGN (pCONT-1) and p50-mKGC (pCONT-2) expression plasmids. Each bar and error bar indicate the average and standard deviation, respectively. The statistical significance between each ORF18 mutant and ORF18 WT was analyzed with one-way ANOVA followed by Tukey’s test. The *p* values are also shown beside the bars. The protein expression levels in the assay are shown in Supplemental Figure S4.

293T cells were co-transfected with plasmids expressing each ORF18 alanine-scanning mutant block [as indicated in the above experiment (Figure 3)] and mCherry expression plasmid (which was used as a transfection control). The transfected cells were subjected to flow cytometry. A red fluoro-protein, mCherry, was used as the transfection control for acquiring more accurate signal intensity. Harvested cells were initially gated by FSC as well as SSC and then mCherry-positive cells were also gated to extract the transfected cells. The ratio of mKG-positive cells was analyzed (Figure 4). The ORF18 block alanine-scanning mutants scoring under 20% were considered as binding-negative mutants because the negative control exhibited approximately 20% scoring in Figure 4a. The substituted residues in ASC2mut, ASC3mut, ASC4mut, ASC5mut, and ASC11mut potentially contained essential residues for ORF30 interaction. The ASC10mut was not included in the list of potential candidates because its protein expression was not confirmed by Western blotting (Supplemental Figure S3b). Another experiment without mCherry showed almost the same results (Supplemental Figure S3a).

Finally, plasmids encoding the ORF18 single alanine-scanning mutants were constructed and subjected to a BiFC interaction assay (Figure 4b). Every single conserved residue in the blocks of ASC2–5mut and ASC10–11mut was substituted with alanine. We included the ASC10mut in this analysis because the ASC10mut expression was deficient in transfected cells. Therefore, it was necessary to evaluate the residues in the ASC10mut block. ORF18 single alanine-scanning mutants scoring under 60% were considered binding-negative mutants because the positive control and most mutants exhibited over 90% scoring. These serial results indicated that Leu(L)29, Glu(E)36, His(H)41, and Trp(W)170 were the responsible residues for ORF30-binding. On the other hand, affinity decreases were not observed in the single mutations of the ASC2 and ASC10 blocks [Pro(P)14, Gly(G)15, and Leu(L)19 of ASC2 and Pro(P)156, Arg(R)158, and Leu(L)159 of ASC10].

**Figure 5:**
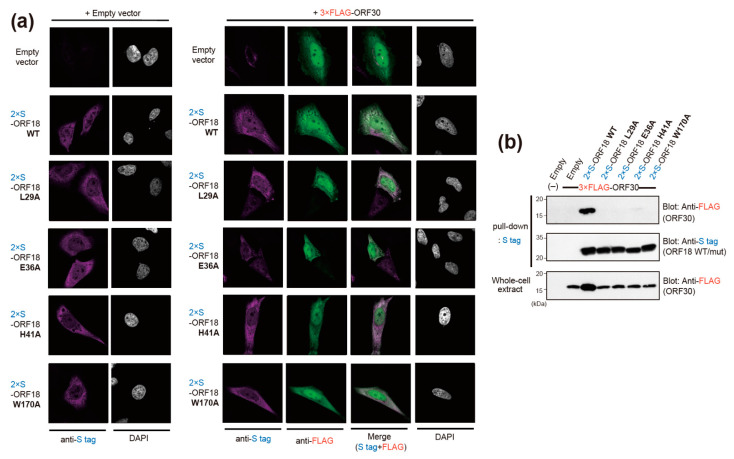
Cellular localization and interaction of ORF18 WT, ORF18 single alanine-scanning mutants, and ORF30 WT. (**a**) HeLa cells were transfected with the indicated expression plasmids or empty vector and then subjected to IFA. Immunofluorescent images were obtained with an inverted confocal microscope. The nuclear DNA was visualized with DAPI staining. 2×S-tagged ORF18 WT or mutants and 3×FLAG-tagged ORF30 are shown in magenta and green, respectively. The DAPI staining is shown in gray. “Merge” indicates the overlaid images of ORF18 (magenta) and ORF30 (green). (**b**) 293T cells were transfected with 2×S-tagged ORF18 WT or mutants, 3×FLAG-tagged ORF30, and/or empty vector as indicated. The transfected cells were lysed and precipitated with S-protein-immobilized agarose beads. The pull-down samples and whole-cell extracts were subjected to western blotting. The original images of the blotting data are shown in Supplementary Figure S10.

Additionally, we confirmed the cellular localization of ORF18 WT and its ORF30-binding deficient single alanine-scanning mutants in the absence or presence of ORF30 by immunofluorescence imaging. HeLa cells were transiently transfected with each epitope-tagged ORF18 and ORF30 expression plasmids as indicated in Figure 5a and were subjected to immunofluorescence analysis (IFA). We found that the ORF18 mutants, ORF18 WT, and ORF30 were localized to both the nucleus and cytoplasm. The ORF18 proteins were comparatively distributed in the cytoplasm, whereas ORF30 was comparatively distributed in the nucleus. A clear co-localization signal of ORF18 WT-ORF30 in the nucleus was not detected, whereas a co-localization signal of ORF18 WT-ORF30 in the cytoplasm was observed. The distribution tendencies of ORF18 mutants-ORF30 have similarities. In order to obtain more direct evidence of the interaction of ORF30 with the ORF18 mutants, we performed pull-down experiments. Our data indicated that ORF30 interacted with ORF18 WT, while interactions of ORF30 with ORF18 mutants (L29A, E36A, H41A, and W170A) were not detected. These pull-down assay results are consistent with the data obtained by our BiFC assay.

### 2.5. AI-Predicted Structure Model of ORF18 and the Identified Residues That Mediate Its Interaction with ORF30

**Figure 6:**
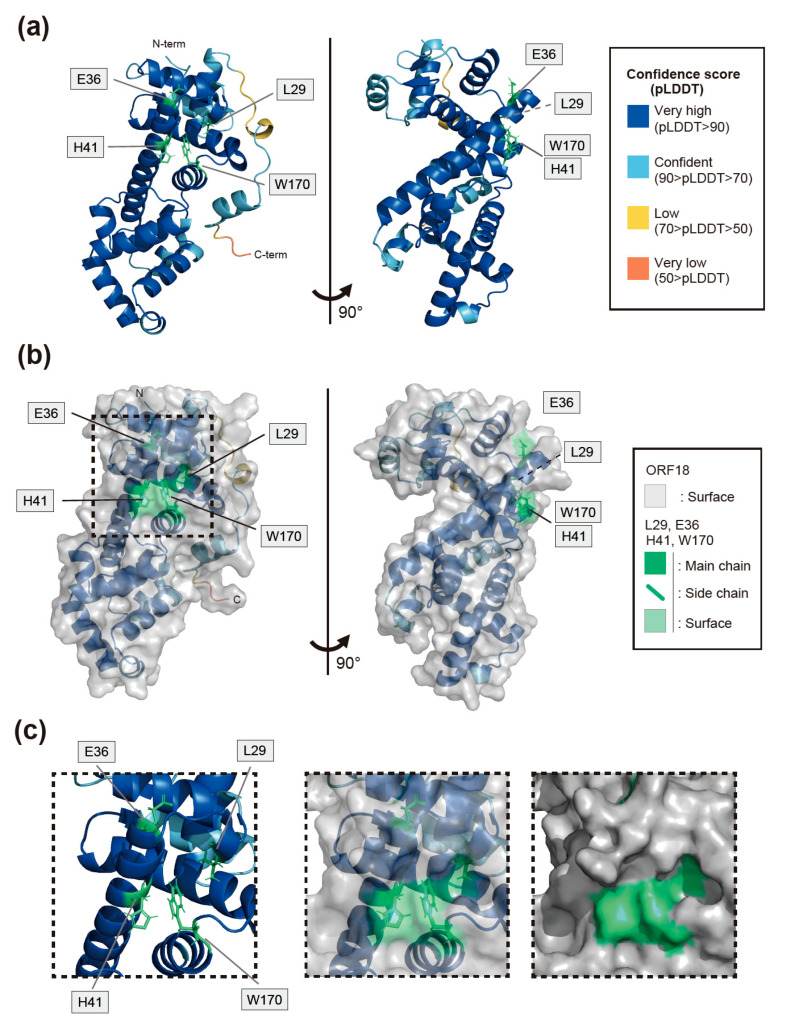
The predicted structure of ORF18 using the AlphaFold2 AI system with the ORF18 amino-acid residues responsible for the interaction with ORF30, as identified with a BiFC assay. (**a**) The whole predicted structure of ORF18 is shown as a cartoon model and two side angles. The cartoon color (blue to orange) indicates the confidence score (pLDDT) calculated by AlphaFold2. The L29, E36, H41, and W170 amino-acid side chains are shown as green stick models. (**b**) Two side views of the cartoon model and stick models in (**a**) were superimposed with the protein surface shown in gray. The surface-exposed L29, E36, H41, and W170 amino-acid side chains were visualized by green transparent shading. The square dotted line area indicates the enclosed regions in Figure 5c. (**c**) The region enclosed within the dotted line in (**b**) is shown. The positions of the L29, E36, H41, and W170 amino-acid residues in the predicted model were enclosed with a cartoon (**right panel**), cartoon/transparent surface (**center panel**), and surface model (**left panel**). Various angles of the predicted models of ORF18 are shown in Supplemental Figure S6.

**Figure 7:**
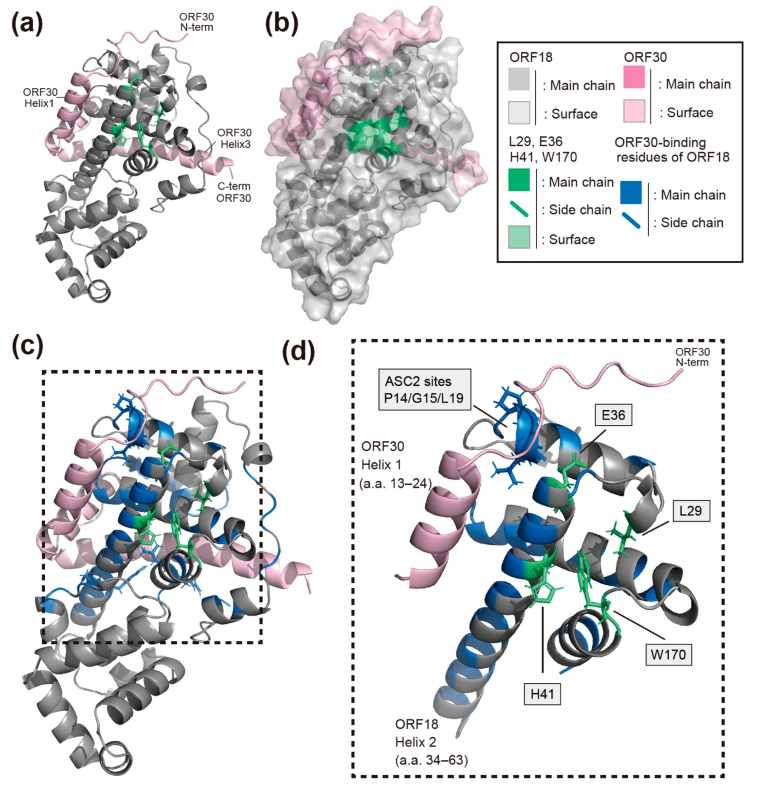
The predicted ORF18-ORF30 complex structure using the multimer prediction mode of AlphaFold2. (**a**) The predicted model is shown as a cartoon. The L29, E36, H41, and W170 amino-acid residues were visualized by green stick models. (**b**) The protein surface was superimposed onto the model shown in Figure 6a. The ORF18 and ORF30 surfaces were visualized by transparent gray and pink shading, respectively. (**c**) The ORF18 amino-acid residues in contact with ORF30 are shown in blue. The square dotted line area indicates the enclosed regions in Figure 6d. (**d**) The extracted structures are in close proximity to ORF30 helix1. The ASC2 mutation target residues (P14/G15/L19) are indicated as blue stick models. The L29, E36, H41, and W170 are indicated by green stick models. Various angles of the predicted ORF18-ORF30 complex with several markers are shown in Supplemental Figures S7–S9.

We attempted to predict the whole protein structure of KSHV ORF18 in order to gain further insight into the function of the four amino-acid residues (L29, E36, H41, and W170) on ORF18 in the interaction with ORF30. To construct a structural model of ORF18, we used the AI deep learning algorithm AlphaFold2. Although we had no prior proof of the utility of AlphaFold2 to predict viral proteins, a predicted structure of KSHV ORF18 was obtained with a high confidence score (Figure 6a). This predicted structure model may be used to evaluate the biological characteristics of the interaction between ORF18 and ORF30. The ORF18 model suggested that all four amino-acid residues which we identified as being responsible for ORF30-binding are exposed to the surface of ORF18 and are located in proximity to each other. In particular, H41, W170, and L29 are in very close proximity and form a straight line on the predicted model surface of ORF18. Although E36 is not located on this H41-W170-L29-straight line, E36 and H41 are closely located on the same helix2 of ORF18 (amino-acid 34–63) (Figure 6a–c and Supplemental Figure S6). Thus, our data obtained with the BiFC assay generally supported the correctness of the AI-predicted structural model. Furthermore, we predicted the ORF18-ORF30 complex model using AlphaFold2. AlphaFold2 has been frequently updated, and a multimer prediction algorithm has recently been implemented. The utilization of a multimer model is not yet fully established. Therefore, the ORF18-ORF30 complex model is introduced in the discussion section (Figure 7a–d and Supplemental Figures S7–S9). 

## 3. Discussion

In this study, four amino-acid residues of KSHV ORF18 required for its binding to ORF30 were identified using a BiFC assay (Figure 2, Figure 3 and Figure 4). Additionally, we utilized the AI deep learning algorithm to predict the structure of the viral protein ORF18 without wet structural data (e.g., X-ray crystallography, NMR, and Cryo-TM). The predicted KSHV ORF18 model, which has a high confidence score, demonstrated that the four identified amino-acid residues responsible for the ORF18-ORF30 interaction were located close together on the surface of ORF18 (Figure 6a–c and Supplemental Figure S6). The AI-predicted structural models of the ORF18 single-molecule and ORF18-ORF30 complex generally supported the molecular interaction data that was obtained with the BiFC assay.

Previous studies have shown that ORF18 and its beta-/gammaherpesvirus homologs are essential for viral replication [21,22,23,24,25]. Furthermore, the amino-acid residues responsible for KSHV ORF18 function have been analyzed and identified [14]. Several ORF18 amino-acid residues, including L29, E36, and W170, are involved in its binding to ORF30 [14]. These three previously reported residues are consistent with the data from our study. These consistent results confirmed the utility of using the BiFC assay to analyze vPIC interactions. However, several interactions between ORF18 and other vPIC factors (e.g., ORF18-ORF34, ORF18-ORF66, and ORF18-ORF31) reported in previous studies, which were analyzed via split-luciferase [11] and pull-down/immunoprecipitation assays [12,14], were not observed with our BiFC assay. Thus, this method may exhibit low sensitivity for weak interactions. Our serial strategy to determine the ORF30-binding site on ORF18 identified an additional novel amino-acid residue, H41 that was required for the ORF18-ORF30 interaction. In order to validate the ORF18-ORF30 interaction data obtained with the BiFC assay, we conducted pull-down experiments. ORF18 WT interacted with ORF30, while ORF30 did not interact with the four ORF18 mutants (ORF18 L29A, E36A, H41A, and W170A), which supported the interaction data obtained with the BiFC assay (Figure 5b). In addition, we found that co-expression of ORF30 and ORF18 WT increased the intracellular expression level of ORF30, whereas ORF18 mutants (L29A, E36A, H41A, W170A) did not (Figure 5b lower panel). These results were also consistent with a previous study [14]. Taken together, these data suggested that the ORF18-ORF30 interaction might contribute to the stability of the ORF30 protein.

ORF18, ORF30, and other KSHV proteins accumulate in the nucleus prior to the formation of the vPIC. However, ORF18 and ORF30 do not have a typical nuclear localization signal (NLS). In order to reveal the mechanism of vPIC formation, we analyzed the localization of ORF18 alone or ORF18 together with ORF30 (Figure 5a). IFA data revealed that the ORF18 mutants, ORF18 WT, and ORF30 were localized in both the nucleus and cytoplasm. Small proteins are known to pass through the nuclear pores by passive diffusion. The native molecular weights of ORF18 and ORF30 are approximately 30 kDa and approximately 9 kDa, respectively. Therefore, ORF18 and ORF30 may be distributed in the nucleus and cytoplasm. However, ORF18 was comparatively distributed in the cytoplasm, whereas ORF30 was comparatively distributed in the nucleus. We did not find a clear co-localization of ORF18 WT-ORF30 in the nucleus, but ORF18 WT and ORF30 appeared to be partially colocalized in the nucleus. These results suggested that the nuclear recruiting or anchoring factor of the ORF18 or ORF18-ORF30 complex is likely another vPIC component or the whole vPIC formation. This is an important issue that should be clarified in future studies.

ORF18 H41 residue was likely detected because our single alanine substitution targets did not only include the amino-acid residues conserved among betaherpesviruses and gammaherpesviruses, but also among KSHV, MHV68, and EBV. Our BiFC experiments revealed two additional observations. First of all, the sequentially conserved amino-acid residues (P14/G15/L19 residues on the ASC2mut target) are responsible for the interaction of ORF18-ORF30, but mutation of each single amino-acid had no effect on the ORF18-ORF30 interaction (Figure 4a,b). This result indicated that the partial structure of P14/G15/L19 residues in ORF18 contributed to its interaction with ORF30. Secondly, we did not detect any protein expression from the Ala-substituted mutant ASC10mut (P156A/R158A/L159A). It is not clear whether the lack of protein expression was due to a direct effect such as destabilization of the protein or an indirect effect such as reduced binding to ORF30 that resulted in a destabilization of the protein. Each single Ala-substituted mutation of P156, R158, or L159 expresses protein and retains the ORF30-binding ability. The requirement of several ORF18 mutations for the loss of ORF30-binding is speculated to provide ORF18 with a safety margin against the failure of binding due to a single missense mutation that may occur upon viral genome replication.

Our AlphaFold2 prediction model of KSHV ORF18 showed that the newly identified H41 residue is located on the same helix (helix2) as the previously identified E36 residue and directly contacts the previously identified W170 residue (Figure 6a–c and Supplemental Figure S6) [14]. Additionally, the AlphaFold2 prediction model of the KSHV ORF18-ORF30 complex revealed several insights into the interaction mode of this complex (Figure 7a–d and Supplemental Figures S7–S9). The predicted model showed that the three identified residues (L29, H41, and W170) of ORF18 are not located on the ORF30-binding surface and are instead located on the opposite side of the ORF30-binding surface. Therefore, it was hypothesized that these three residues do not directly form the ORF30-binding surface structure, but instead, these residues contribute to forming the scaffold of the ORF30-binding surface. KSHV ORF30 is a short-length protein, and it is predicted to contain three helices. The model showed that all three helices of ORF30 fill the groove of ORF18 when ORF30 is bound to ORF18. The ORF30 N-terminus and helix1 (amino-acid 13–24) are estimated to be in proximity to the four identified residues of ORF18 (L29, E36, H41, and W170) that are required for the ORF18-ORF30 interaction (Figure 7a,c,d). The ORF18 helix2 harboring E36 and H41 is located along and parallel with the ORF30 helix1. E36 is located on ORF18 helix2 and directly contacts the N-terminal tail of ORF30, but H41 does not contact the ORF30 helix1. However, it is possible that H41 contributes to the proper orientation of both ORF18 helix2 and ORF30 helix1. The H41 in ORF18 forms a straight line with W170 and L29, which may support the ORF18 helix2 as a scaffold (Figure 7d). Direct contact of P14/G15/L19 (ASC2mut target) to the ORF30 N-terminal tail was also observed. The combined results of the AI prediction model and BiFC data provided further insight regarding the interaction residues of N53/Y55 (partial ASC6mut target) and R158/L159 (partial ASC10mut target). These residues may be involved in supporting the ORF30 helix3 (Supplemental Figure S9). The ORF18 ASC6mut exhibited mildly decreased binding to ORF30 and the ORF18 ASC10mut protein was not expressed in transfected cells (Figure 4a,b). The importance and interaction characteristics of E36 and R158/L159 were already reported [14] and agree well with our experimental data and prediction model. However, in order to reveal the real interactions between ORF18 and ORF30, characterization by X-ray crystallography is required.

AI-prediction protein modeling has been used in our vPIC analysis, for the assessment of the appropriateness of the real experimental data observed by pull-down and Immuno-precipitation assays [14]. It has been accepted that homology modeling is a useful method for predicting protein structure. However, the prediction of viral proteins that lack related homologous structures may not be reliable because of the subjective nature of selecting the specific protein based on the prediction. Expansion of protein modeling in silico may lead to a paradigm shift in the study of protein structure in various life science fields, including virology. With the exclusion of intensely studied viruses, a massive amount of viral protein structures remains unknown. Thus, these innovations would enhance the data-driven study of the viral proteins whose structure is unknown.

## 4. Materials and Methods

### 4.1. Cell Culture and Reagents

293T and HeLa cells (RCB2202 and RCB0007 respectively; RIKEN Bio Resource Center, Tsukuba, Japan) were cultured in DMEM supplemented with 10% fetal calf serum. The transfection reagent, PEI-MAX MW40000 (Polysciences, Inc., Warrington, PA, USA) was dissolved at a concentration of 2 mg/mL in distilled water and filtered.

### 4.2. Plasmids

KSHV ORF18, ORF24, ORF30, ORF34, and ORF66 coding fragments were obtained by PCR from KSHV BAC16 (GQ994935; KSHV BAC clone based on strain JSC-1) [26] using primer sets noted in the Supplemental Table S1. These fragments were cloned into the phmKGN-MC, phmKGC-MC, phmKGN-MN, and phmKGC-MN vectors. These empty backbone vectors were included in the CoralHue™ Fluo-chase Kit (Medical & Biological Laboratories Co., Ltd., Tokyo, Japan). The KSHV ORF18 truncated mutants and alanine-scanning mutant coding fragments were obtained by 2-step PCR using primer sets noted in the Supplemental Table S1 and they were cloned into the phmKGN-MC vector. The mCherry coding fragment was obtained by PCR from pEF6.mCherry-TSG101 [27] (Addgene plasmid # 38318; gifted from Dr. Quan Lu) using primer sets noted in the Supplemental Table S1 and it was cloned into the pCI-neo mammalian expression vector (Promega, Madison, WI, USA). The KSHV ORF18 WT and single alanine-scanning mutants (L29A, E36A, H41A, and W170A) were cloned by PCR from ph-mKGN-ORF18 WT, L29A, E36A, H41A, and W170A plasmids using primer sets noted in the Supplemental Table S1. The PCR products were then cloned into the pCIneo-2×S vector. The pCIneo-3×FLAG-ORF30 expression plasmids were constructed in our previous report [12].

### 4.3. BiFC Interaction Assay

Our BiFC assays were conducted according to a previous method reported by Yoshida et al. [28]. Briefly, 3 × 10^5^ of 293T cells were seeded into each well of a 12 well plate. After an overnight incubation, the cells were transfected with mKGN-tagged protein expression plasmids and mKGC-tagged protein expression plasmids. The calcium phosphate method was used to transfect a total of 3 μg of plasmid (1.5 μg of each expression plasmid). The PEI (Polyethylenimine) method was also used to transfect a total of 1.6 mg of plasmid (0.8 μg of each expression plasmid or 0.5 μg of each expression plasmid and 0.6 μg of mCherry expression plasmid, which was utilized as a transfection control). After two days following the transfection, the cells were harvested and fixed with 1% formaldehyde in PBS and subjected to flow-cytometry analysis. In order to confirm the protein expression, cells transfected in parallel were used to prepare protein extracts, which were subjected to Western blotting.

### 4.4. Western Blotting and Pull-Down Assay

Western blotting and pull-down assay were performed as described previously [12,15]. Anti-monomeric Kusabira-Green N-terminal fragment antibodies (M148-3M), anti-DDDDK-tag monoclonal antibodies (FLA-1), and anti-S tag polyclonal antibodies were purchased from Medical & Biological Laboratories Co., Ltd., and anti-actin antibodies (AC-15) was obtained from Santa-Cruz Biotechnology, Inc. (Dallas, TX, USA). HRP linked anti-mouse IgG antibody (GE healthcare UK Ltd., Buckinghamshire, UK or Jackson ImmunoResearch, Inc., West Grove, PA, USA) and HRP linked anti-rabbit IgG antibody (Jackson ImmunoResearch, Inc.) were used as the secondary antibody. Antibody-bound proteins were visualized with ECL Western Blotting Detection Reagents (GE healthcare UK Ltd. or ATTO, Tokyo, Japan) on X-ray film (Fujifilm-Wako, Tokyo, Japan). For pull-down assays, transfected 293T cells were lysed by brief sonication in the presence of lysis buffer [20 mM HEPES (pH 7.9), 0.18 M NaCl, 0.1% NP-40, 0.1 mM EDTA, 10% glycerol, and cOmplete, Mini, EDTA-free Protease Inhibitor Cocktail (Roche Diagnostics GmbH, Mannheim, Germany)]. The cell extracts were subjected to affinity purification using S-protein-agarose (Merck Millipore, Burlington, MA, USA). Purified proteins and whole-cell extracts were subjected to western blotting.

### 4.5. Immunofluorescence Analysis (IFA)

IFA was performed as described previously with several modifications [12]. HeLa cells were seeded and co-transfected with pCI-neo empty vector, pCIneo-3×FLAG-ORF30, and pCIneo-2×S-ORF18 WT/single alanine-scanning mutant as indicated using the PEI-Max reagent. After 48 h, the transfected cells were seeded on coverslips. The following day, the cells were fixed with 4% p-formaldehyde/PBS at room temperature for 10 min, and sequentially incubated with ice-cold methanol at room temperature for 10 min. After washing with PBS, the cells were blocked with 1% BSA/0.3 M glycine/PBS and then incubated at room temperature for 1.5 h with Anti-DDDDK-tag monoclonal antibodies (dilution rate 1:1000) and anti-S tag polyclonal antibodies (1:250). After washing with PBS, the cells were further incubated at room temperature for 1 h with fluorescein isothiocyanate (FITC) conjugated anti-mouse IgG (1:2000) (Rockland Immunochemicals Inc., Pottstown, PA, USA) and Cy3 conjugated anti-rabbit IgG (1:2000) (Fujifilm-Wako). After washing with PBS, the stained cells attached to coverslips were mounted on glass slides using ProLong Diamond Antifade Mountant with DAPI (Thermo Fisher Scientific, Waltham, MA, USA). Immunofluorescent images were obtained with an inverted confocal microscope (Nikon C2; Nikon, Tokyo, Japan). The raw images were processed with the NIS-Elements Viewer software (ver 5.21.00; Nikon) using Auto-LUTs. The images exhibiting red color in the Cy3 channel were converted to magenta using Adobe Photoshop software for universal color design.

### 4.6. Statistics

The standard deviation was determined by analyzing the data obtained from three independent samples and is indicated as the error bars. The statistical difference between the negative controls/ORF18 WT and each sample, noted in each figure legend, was analyzed by one-way ANOVA followed by Tukey’s test for multiple comparison using R (ver 4.0.3) and R Studio. The *p* values are shown in each figure.

### 4.7. Protein Structure Prediction

The KSHV ORF18 protein structure model and the KSHV ORF18-ORF30 complex model were predicted using AlphaFold 2.1.2 and 2.2.0 (https://github.com/deepmind/alphafold (accessed on 20 Feburuary 2022, 31 March 2022, respectively)) in its own local environment, respectively [20]. The following databases were utilized for AlphaFold2: Uniclust30 (version 2018_08), MGnify (version 2018_12), pdb70 (downloaded on 15 February 2022), PDB/mmCIF (downloaded on 18 February 2022), and pdb_seqres (downloaded on 18 February 2022). It is noted that several versions or frequently updated versions of these databases were utilized. The predicted model was visualized with the molecular visualization open-source software, PyMOL (ver 2.5.0). In addition, the pLDDT score coloring of the protein model was visualized with the python module PSICO (https://github.com/YoshitakaMo/pymol-psico, https://github.com/speleo3/pymol-psico (accessed on 7 April 2022)).

## 5. Conclusions

We analyzed the interactions between KSHV ORF18 and the other components (ORF24, ORF30, ORF31, ORF34, and ORF66) of vPIC in living cells using a BiFC assay. As a result, an apparent BiFC-positive signal was detected only between ORF18 and ORF30. The four amino-acid residues (L29, E36, H41, and W170) in ORF18 were necessary for its interaction with ORF30. Moreover, the AI-predicted structure model of ORF18 suggested that these amino-acid residues are in proximity to each other and form the surface structure that is required for ORF18 binding to ORF30. Thus, the AI-predicted model supported the importance of these four residues for binding ORF18 to ORF30.

## Data Availability

Plasmid DNA sequence data are available from the corresponding author upon reasonable request. Other data are presented in the manuscript.

## Supplementary Materials

The following supporting information can be downloaded at: https://www.mdpi.com/article/10.3390/ijms23179647/s1.

## References

[1] Chang Y., Cesarman E., Pessin M.S., Lee F., Culpepper J., Knowles D.M., Moore P.S. (1994). Identification of herpesvirus-like DNA sequences in AIDS-associated Kaposi’s sarcoma. Science.

[2] Nador R.G., Cesarman E., Chadburn A., Dawson D.B., Ansari M.Q., Sald J., Knowles D.M. (1996). Primary effusion lymphoma: A distinct clinicopathologic entity associated with the Kaposi’s sarcoma-associated herpes virus. Blood.

[3] Soulier J., Grollet L., Oksenhendler E., Cacoub P., Cazals-Hatem D., Babinet P., d’Agay M.F., Clauvel J.P., Raphael M., Degos L. (1995). Kaposi’s sarcoma-associated herpesvirus-like DNA sequences in multicentric Castleman’s disease. Blood.

[4] Russo J.J., Bohenzky R.A., Chien M.C., Chen J., Yan M., Maddalena D., Parry J.P., Peruzzi D., Edelman I.S., Chang Y. (1996). Nucleotide sequence of the Kaposi sarcoma-associated herpesvirus (HHV8). Proc. Natl. Acad. Sci. USA.

[5] Sudomova M., Berchova-Bimova K., Mazurakova A., Samec D., Kubatka P., Hassan S.T.S. (2022). Flavonoids target human herpesviruses that infect the nervous system: Mechanisms of action and therapeutic insights. Viruses.

[6] Tso F.Y., Sawyer A., Kwon E.H., Mudenda V., Langford D., Zhou Y., West J., Wood C. (2017). Kaposi’s sarcoma-associated herpesvirus infection of neurons in HIV-Positive patients. J. Infect. Dis..

[7] Baldini F., Baiocchini A., Schininà V., Agrati C., Giancola M.L., Alba L., Grisetti S., Del Nonno F., Capobianchi M.R., Antinori A. (2013). Brain localization of Kaposi’s sarcoma in a patient treated by combination antiretroviral therapy. BMC Infect. Dis..

[8] Jha H.C., Mehta D., Lu J., El-Naccache D., Shukla S.K., Kovacsics C., Kolson D., Robertson E.S. (2015). Gammaherpesvirus Infection of Human Neuronal Cells. mBio.

[9] Krug L.T., Pellet P.E., Damania B.A., Cohen J.I. (2022). The Family Herpesviridae: A Brief Introduction.

[10] Gruffat H., Marchione R., Manet E. (2016). Herpesvirus late gene expression: A viral-specific pre-initiation complex is key. Front. Microbiol..

[11] Davis Z.H., Hesser C., Park J., Glaunsinger B.A. (2015). Interaction between ORF24 and ORF34 in the Kaposi’s sarcoma-associated herpesvirus late gene transcription factor complex is essential for viral late gene expression. J. Virol..

[12] Nishimura M., Watanabe T., Yagi S., Yamanaka T., Fujimuro M. (2017). Kaposi’s sarcoma-associated herpesvirus ORF34 is essential for late gene expression and virus production. Sci. Rep..

[13] Brulois K., Wong L.Y., Lee H.R., Sivadas P., Ensser A., Feng P., Gao S.J., Toth Z., Jung J.U. (2015). Association of Kaposi’s Sarcoma-associated herpesvirus ORF31 with ORF34 and ORF24 is critical for late gene expression. J. Virol..

[14] Castañeda A.F., Glaunsinger B.A. (2019). The Interaction between ORF18 and ORF30 Is Required for Late Gene Expression in Kaposi’s Sarcoma-Associated Herpesvirus. J. Virol..

[15] Watanabe T., Nishimura M., Izumi T., Kuriyama K., Iwaisako Y., Hosokawa K., Takaori-Kondo A., Fujimuro M. (2020). Kaposi’s Sarcoma-associated herpesvirus orf66 is essential for late gene expression and virus production via interaction with ORF34. J. Virol..

[16] Didychuk A.L., Castaneda A.F., Kushnir L.O., Huang C.J., Glaunsinger B.A. (2020). Conserved CxnC Motifs in Kaposi’s Sarcoma-associated herpesvirus ORF66 are required for viral late gene expression and are essential for its interaction with ORF34. J. Virol..

[17] Kodama Y., Hu C.D. (2012). Bimolecular fluorescence complementation (BiFC): A 5-year update and future perspectives. Biotechniques.

[18] David A., Islam S., Tankhilevich E., Sternberg M.J.E. (2022). The AlphaFold database of protein structures: A biologist’s guide. J. Mol. Biol..

[19] Fersht A.R. (2021). AlphaFold—A personal perspective on the impact of machine learning. J. Mol. Biol..

[20] Jumper J., Evans R., Pritzel A., Green T., Figurnov M., Ronneberger O., Tunyasuvunakool K., Bates R., Zidek A., Potapenko A. (2021). Highly accurate protein structure prediction with AlphaFold. Nature.

[21] Arumugaswami V., Wu T.T., Martinez-Guzman D., Jia Q., Deng H., Reyes N., Sun R. (2006). ORF18 is a transfactor that is essential for late gene transcription of a gammaherpesvirus. J. Virol..

[22] Gong D., Wu N.C., Xie Y., Feng J., Tong L., Brulois K.F., Luan H., Du Y., Jung J.U., Wang C.Y. (2014). Kaposi’s sarcoma-associated herpesvirus ORF18 and ORF30 are essential for late gene expression during lytic replication. J. Virol..

[23] Wu T.-T., Park T., Kim H., Tran T., Tong L., Martinez-Guzman D., Reyes N., Deng H., Sun R. (2009). ORF30 and ORF34 are essential for expression of late genes in Murine Gammaherpesvirus 68. J. Virol..

[24] Perng Y.C., Campbell J.A., Lenschow D.J., Yu D. (2014). Human cytomegalovirus pUL79 is an elongation factor of RNA polymerase II for viral gene transcription. PLoS Pathog..

[25] Pan D., Han T., Tang S., Xu W., Bao Q., Sun Y., Xuan B., Qian Z. (2018). Murine Cytomegalovirus Protein pM91 Interacts with pM79 and Is Critical for Viral Late Gene Expression. J. Virol..

[26] Brulois K.F., Chang H., Lee A.S., Ensser A., Wong L.Y., Toth Z., Lee S.H., Lee H.R., Myoung J., Ganem D. (2012). Construction and manipulation of a new Kaposi’s sarcoma-associated herpesvirus bacterial artificial chromosome clone. J. Virol..

[27] Nabhan J.F., Hu R., Oh R.S., Cohen S.N., Lu Q. (2012). Formation and release of arrestin domain-containing protein 1-mediated microvesicles (ARMMs) at plasma membrane by recruitment of TSG101 protein. Proc. Natl. Acad. Sci. USA.

[28] Yoshida T., Ebina H., Koyanagi Y. (2009). N-linked glycan-dependent interaction of CD63 with CXCR4 at the Golgi apparatus induces downregulation of CXCR4. Microbiol. Immunol..

