# Supplementary material for: Biomolecular Fluorescence Complementation Profiling and Artificial Intelligence Structure Prediction of the Kaposi’s Sarcoma-Associated Herpesvirus ORF18 and ORF30 Interaction
Source: Int J Mol Sci. 2022 Aug 25;23(17):9647. doi: 10.3390/ijms23179647 (PMC9456320; doi:10.3390/ijms23179647)
Supplement: Supplementary file 1 [file ijms-23-09647-s001.zip › ijms-1888080-supplementary.pdf]

Supplemental Figure S1

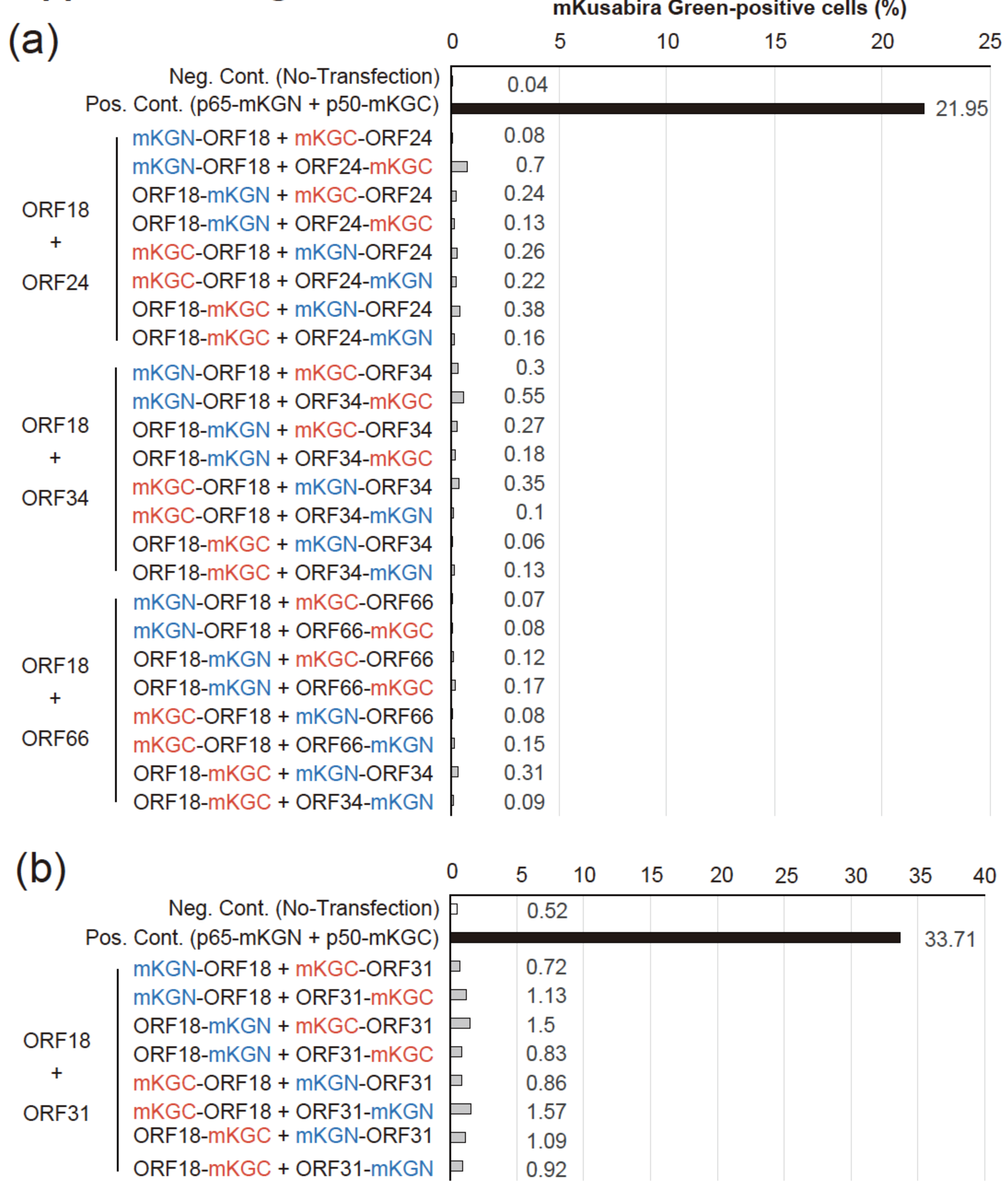

Supplemental Figure S1.

(a and b) Screening and optimization of the BiFC assay to determine vPIC component interactions. Each indicated combination of the expression plasmids were co-transfected into 293T cells by the calcium phosphate method and single samples were assessed by flow cytometry. The negative control (Neg. Cont.) consisted of non-transfected cells, and the positive control (Pos. Cont.) comprised cells co-transfected with p65-mKGN (pCONT-1) and p50-mKGC (pCONT-2) expression plasmids.

Supplemental Figure S2

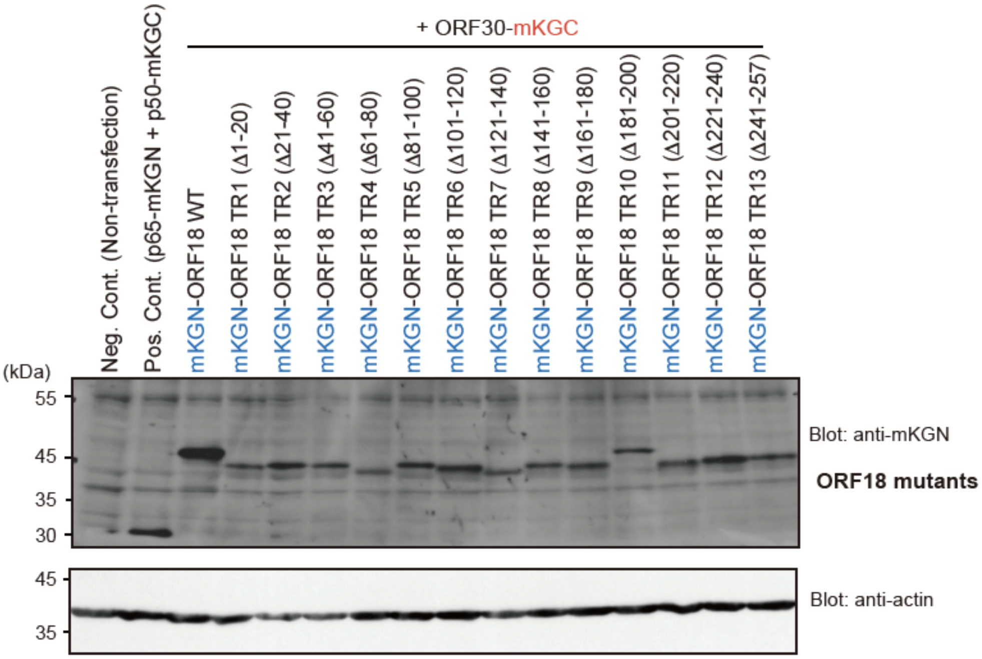

Supplemental Figure S2.

Confirmation of ORF18 truncated mutant protein expression.

Each indicated combination of the expression plasmids were simultaneously and independently co-transfected in order to conduct a BiFC assay (as described in Fig. 2) and a Western blot (described here). The co-transfected cells were lysed and subjected to Western blotting using anti-mKGN primary antibodies. An antibody that recognizes actin (anti-actin) was used as a loading control. The original blotting data are shown in Supplemental Figure S5.

Supplemental Figure S3

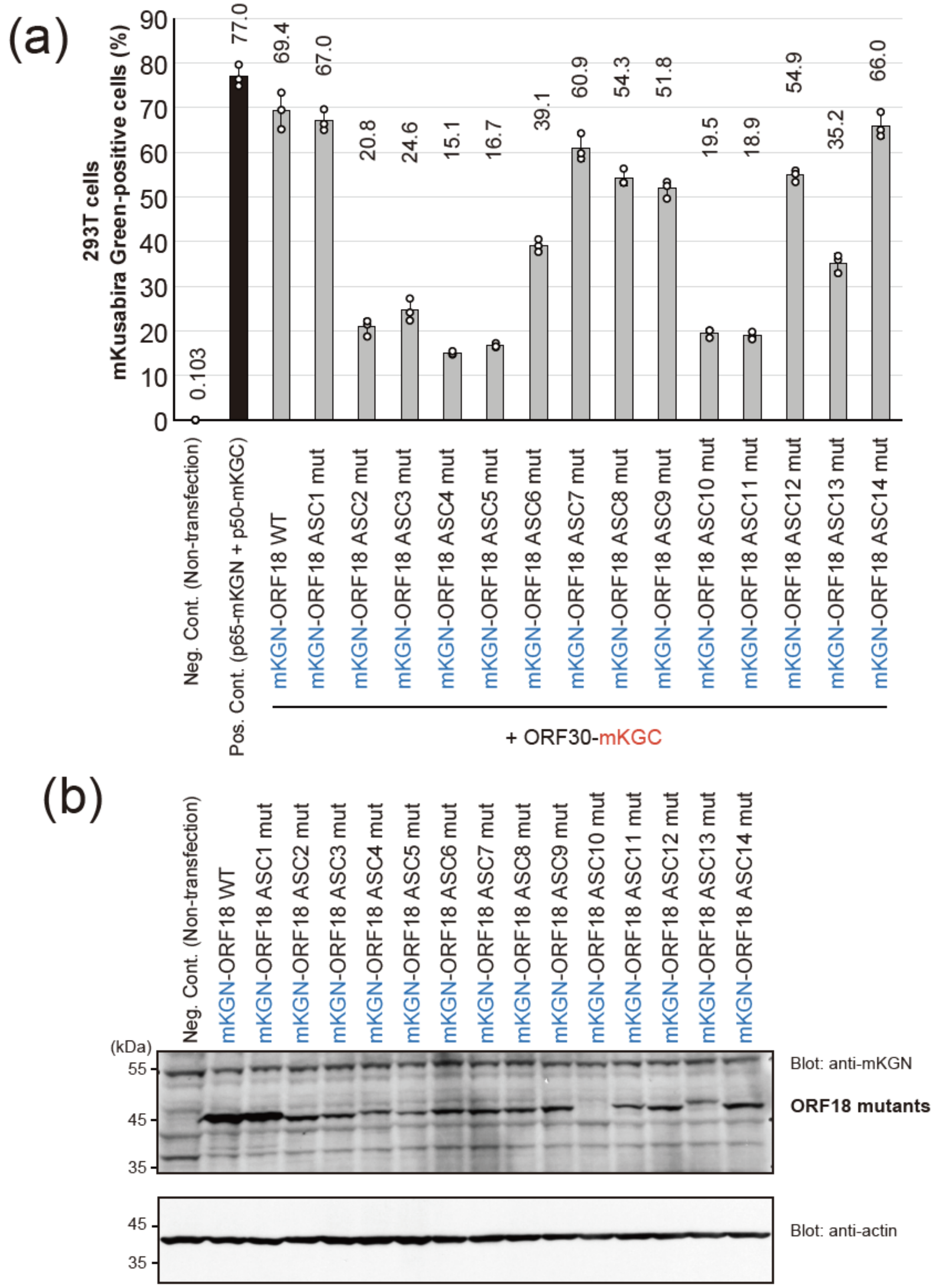

Supplemental Figure S3.

(a) BiFC assay of ORF18 block alanine-scanning mutants (ASC1mut-ASC14mut). Each mKGN-ORF18 block alanine-scanning mutant expression plasmid was co-transfected into 293T cells with ORF30-mKGC. The 293T cells were transfected with a lipofection method and three independent samples were assessed by flow cytometry. The negative control (Neg. Cont.) was non-transfected cells and the positive control (Pos. Cont.) comprised cells co-transfected with p65-mKGN (pCONT-1) and p50-mKGC (pCONT-2). Each bar and error bar indicate the average and standard deviation, respectively.

(b) Protein expression of each ORF18 block alanine-scanning mutant (ASC1mut-ASC14mut). Each mKGN-ORF18 block alanine-scanning mutant expression plasmid was co-transfected into 293T cells with ORF30-mKGC. The samples were subjected to Western blotting using anti-mKGN primary antibody. An antibody that recognizes actin (anti-actin) was used as a loading control. The original blotting data are shown in Supplemental Figure S5.

Supplemental Figure S4

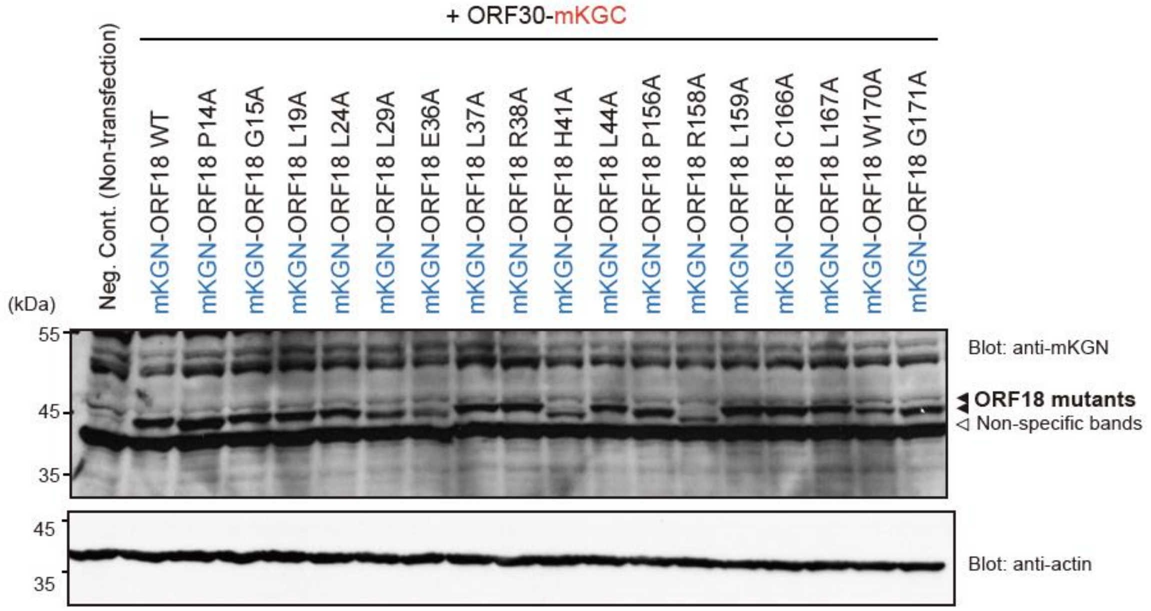

Supplemental Figure S4.

S4. Protein expression of each ORF18 single alanine mutant. Each indicated combination of the expression plasmids were simultaneously and independently co-transfected in order to conduct a BiFC assay (as described in Fig. 4b) and a Western blot (described here). The cells were lysed and subjected to Western blotting using anti-mKGN primary antibodies. An antibody that recognizes actin (anti-actin) was used as a loading control. The original blotting data are shown in Supplemental Figure S5.

Supplemental Figure S5

(a)

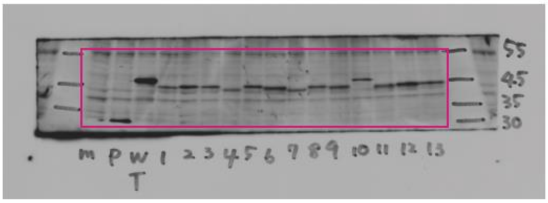

(b)

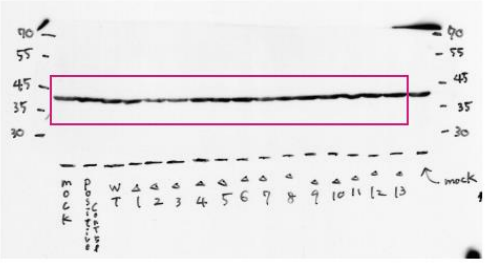

(c)

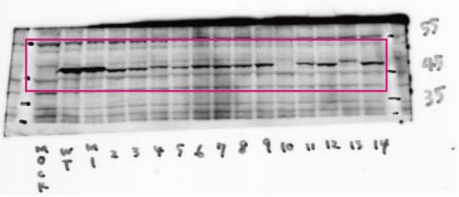

(d)

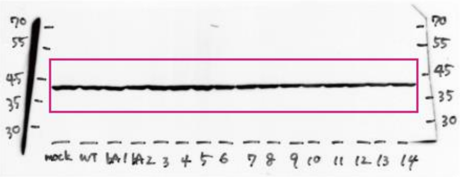

(e)

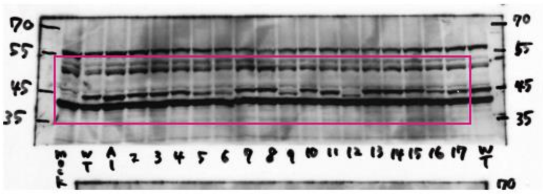

(f)

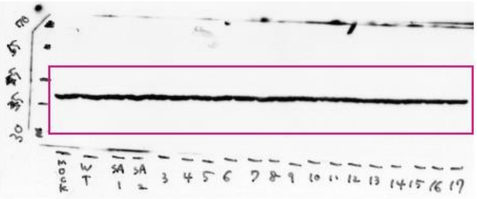

Supplemental Figure S5

Original Western blotting data (X-ray film) of Supplemental Figure S2, S3, and S4.

(a) Original anti-mKGN blotting data of Supplemental Figure S2 upper panel.

(b) Original anti-actin blotting data of Supplemental Figure S2 lower panel.

(c) Original anti-mKGN blotting data of Supplemental Figure S3b upper panel.

(d) Original anti-actin blotting data of Supplemental Figure S3b lower panel.

(e) Original anti-mKGN blotting data of Supplemental Figure S4 upper panel.

(f) Original anti-actin blotting data of Supplemental Figure S4 lower panel.

Supplemental Figure S6

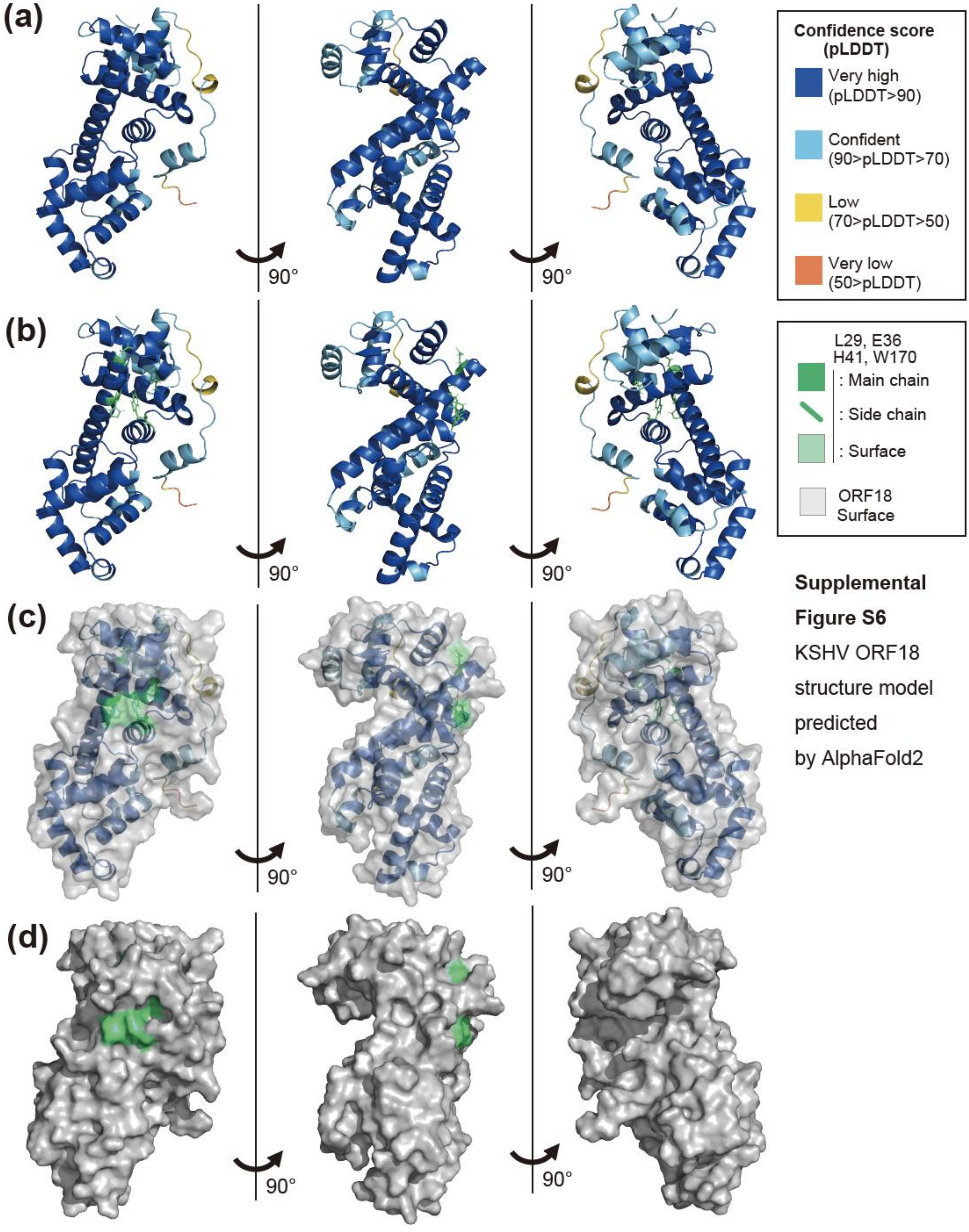

Supplemental Figure S6
KSHV ORF18 structure model predicted by AlphaFold2

Supplemental Figure S7

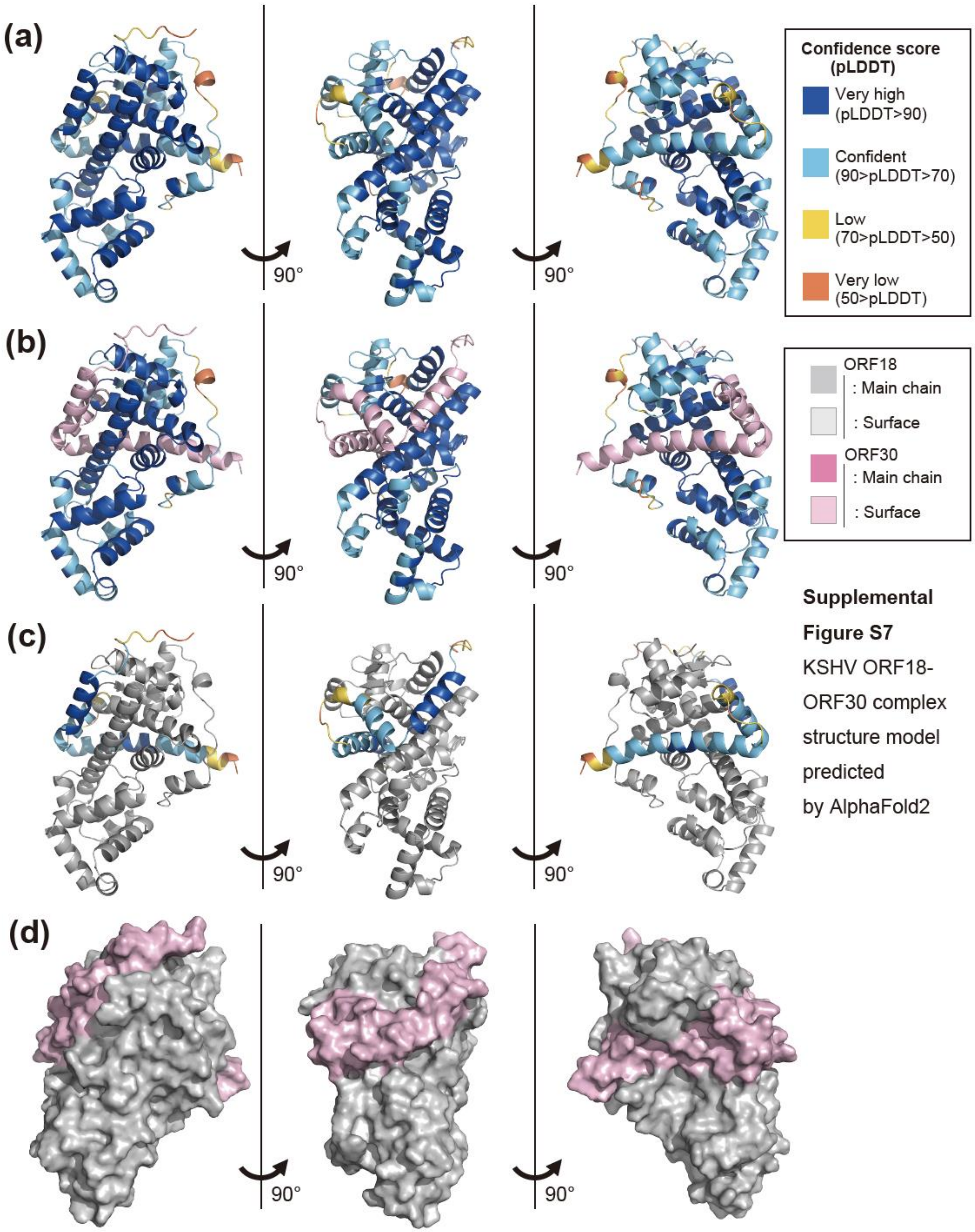

Supplemental Figure S7
KSHV ORF18-ORF30 complex structure model predicted by AlphaFold2

Supplemental Figure S8

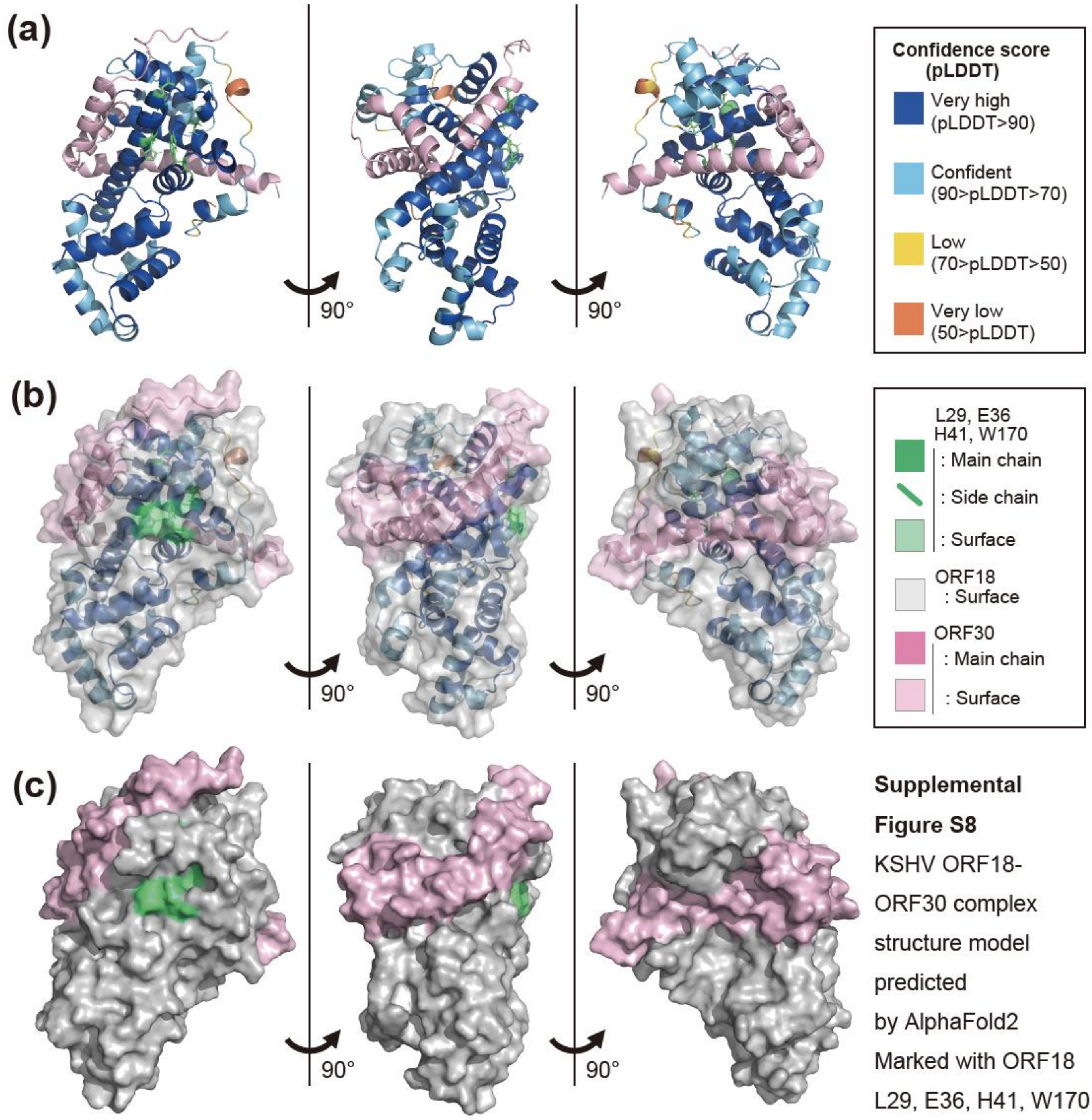

Supplemental Figure S8
KSHV ORF18-ORF30 complex structure model predicted by AlphaFold2 Marked with ORF18 L29, E36, H41, W170

Supplemental Figure S9

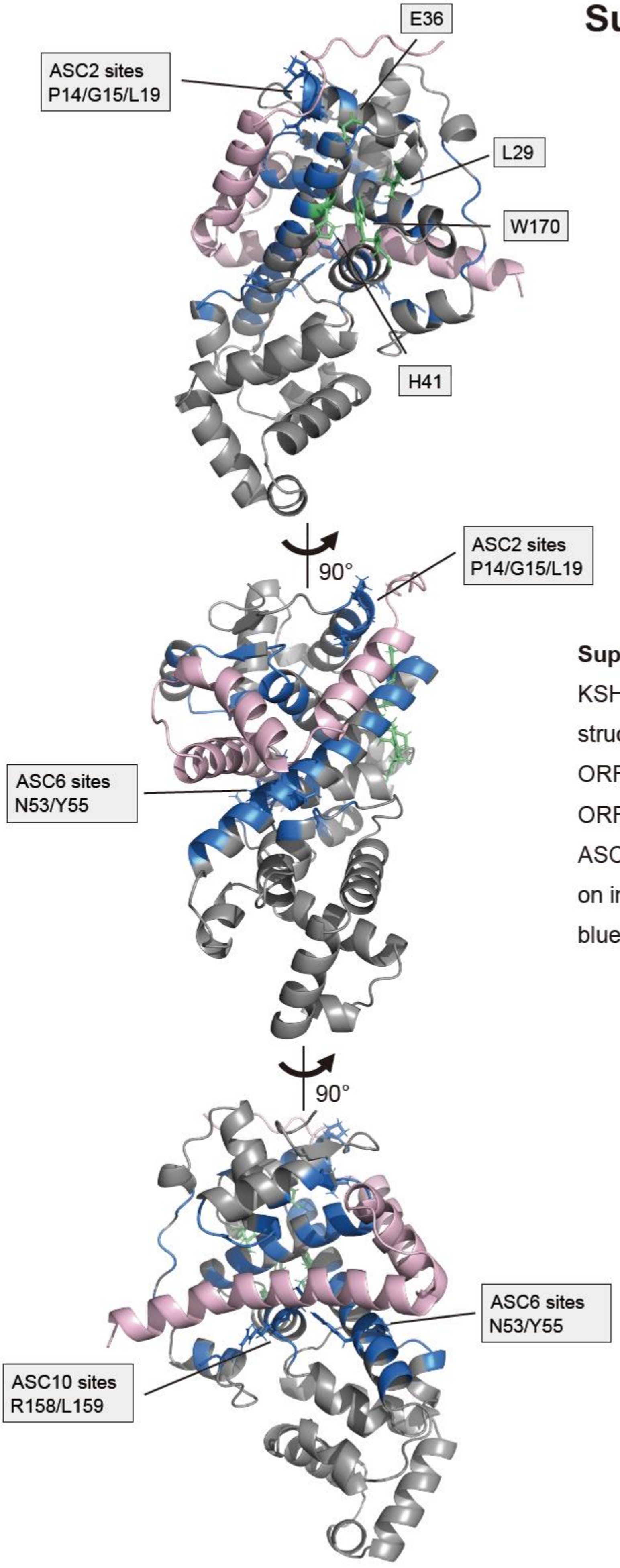

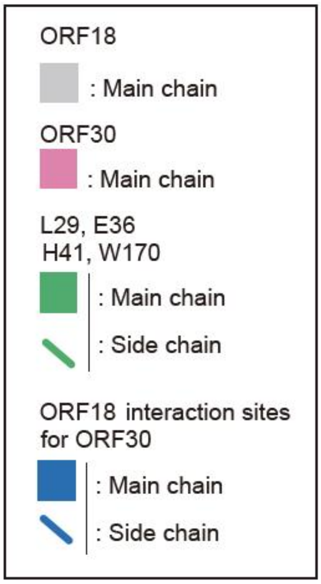

Supplemental Figure S9

KSHV ORF18- ORF30 complex structure model predicted by AlphaFold2; ORF18 interaction residues for ORF30 were marked with blue cartoon. ASC mutants (Figure5a) target amino-acids on interaction residues are emphasized as blue sticks and tags.

Supplemental Figure S10

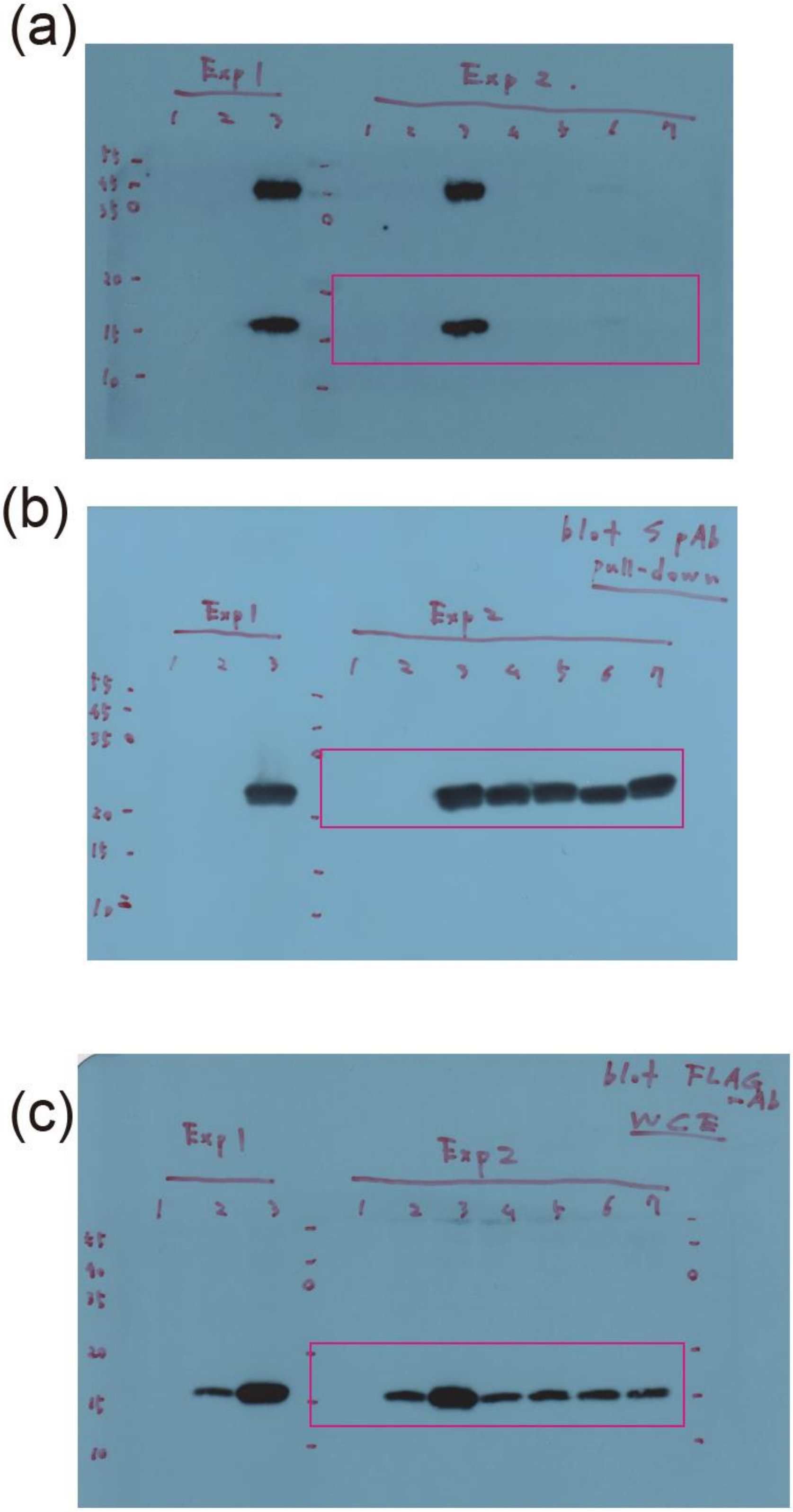

Supplemental Figure S10

Original Western blotting data (X-ray film) of Figure 5b

(a) Original anti-FLAG blotting data of Figure 5b upper panel.

(b) Original anti-Stag blotting data of Figure 5b Middle panel.

(c) Original anti-FLAG blotting data of Figure 5b lower panel.

Supplemental TABLE S1: Primers for construction of expression plasmids

Plasmid name	Primer name	Primer sequences (5' -> 3')	
[vPIC components]			
ph-ORF18-mKGN<br>ph-ORF18-mKGC<br>ph-mKGN-ORF18<br>ph-mKGC-ORF18	S2_EcoRI-kshvORF18	catcGAATTCgatgctcggaaaatacgtgtgtgagac	*a
	As(stp)_kshvORF18-NotI	taaGCGGCCGCttaaaccgcgttgttgttaaac	*a
	As2(Nstp)_kshvORF18-NotI	gttGCGGCCGCaaccgcgttgttgttaaac	*a
ph-ORF30-mKGN<br>ph-ORF30-mKGC<br>ph-mKGN-ORF30<br>ph-mKGC-ORF30	S2_EcoRI-kshvORF30	atcGAATTCgatgggtgagccagtggatcctggac	*a
	As(stp)_kshvORF30-NotI	tgaGCGGCCGCtcatttcgcaccggtgtctag	*a
	As2(Nstp)_kshvORF30-NotI	aaaGCGGCCGCtttcgcaccggtgtctaggc	*a
ph-ORF24-mKGN<br>ph-ORF24-mKGC<br>ph-mKGN-ORF24<br>ph-mKGC-ORF24	S_BamHI-kshvORF24	catGGATCCatggcagcgctcgaggg	*a
	As(stp)_kshvORF24-HindIII,	ctaaAAGCTTttagaccagcggacggacgcaac	*a
	As2(Nstp)_kshvORF24-HindIII	gtcAAGCTTgaccagcggacggacgc	*a
ph-ORF31-mKGN<br>ph-ORF31-mKGC<br>ph-mKGN-ORF31<br>ph-mKGC-ORF31	S2_EcoRI-kshvORF31	catcGAATTCgatgtcacaaaacagaaagactctgcctgc	*a
	As(stp)_kshvORF31-NotI	tagGCGGCCGCctacgtatctttcgttgatagcatg	*a
	As2(Nstp)_kshvORF31-NotI	acgGCGGCCGCcgtatctttcgttgatagcatg	*a
ph-ORF34-mKGN<br>ph-ORF34-mKGC<br>ph-mKGN-ORF34<br>ph-mKGC-ORF34	S_XhoI-kshvORF34	cattCTCGAGaatgtttgctttgagctcgc	*a
	As(stp)_kshvORF34-HindIII	ctaaAAGCTTtagagttggttgagtccattctcc	*a
	As2(Nstp)_kshvORF34-HindIII	actcAAGCTTgagttggttgagtccattctc	*a
ph-ORF66-mKGN<br>ph-ORF66-mKGC<br>ph-mKGN-ORF66<br>ph-mKGC-ORF66	S_XhoI-kshvORF66	cattCTCGAGaatggccctggatcagcgct	*a
	As(stp)_kshvORF66-HindIII	ctgaAAGCTTtcaggaggaacacttcccgcaacag	*a
	As2(Nstp)_kshvORF66-HindIII	ctccAAGCTTggaggaacacttcccgcaac	*a
[Expression control plasmids]			
pCIneo-mCherry	S_EcoRI_mCherry	catcGAATTCgatggtgagcaagggcgag	*a
	As(stp)_mCherry_SalI	tagGTCGACctacttgtacagctcgtccatg	*a
[ORF18 single mutants]			
ph-mKGN-ORF18-P14A	S_1_KSHV_ORF18_P14A	ccgctgagcGCAgggcttaggaggctcatg	*b
	As_ 1_KSHV_ORF18_P14A	ctaagcccTGCgctcagcggctcggtctc	*b
ph-mKGN-ORF18-G15A	S_2_ KSHV_ORF18_G15A	ctgagcccgGCGcttaggaggctcatgtgg	*b
	As_2_ KSHV_ORF18_G15A	ctcctaagCGCcgggctcagcggctcggtc	*b
ph-mKGN-ORF18-L19A	S_3_ KSHV_ORF18_L19A	gcttaggaggGCCatgtggcgctttttgcaaaataag	*b
	As_3_ KSHV_ORF18_L19A	gccacatGGCcctcctaagccccgggctc	*b
ph-mKGN-ORF18-L24A	S_4_ KSHV_ORF18_L24A	gtggcgctttGCAcaaaataagaatttaaatac	*b
	AS_4_ KSHV_ORF18_L24A	cttattttgTGCaaagcgccacatgagcctc	*b
ph-mKGN-ORF18-L29A	S_5_KSHV_ORF18_L29A	gaatGCAaatacattccacgcccaag	*b
	As_5_KSHV_ORF18_L29A	cgtggaatgtattTGCattcttattttgcaaaaag	*b
ph-mKGN-ORF18-E36A	S_6_KSHV_ORF18_E36A	cattccacgcccaaGCActgcgttttattcatttg	*b
	As_6_KSHV_ORF18_E36A	cagTGCttgggcgtggaatgtatttaaattc	*b
ph-mKGN-ORF18-L37A	S_7_KSHV_ORF18_L37A	ccaagagGCCcgttttattcatttggttc	*b
	As_7_KSHV_ORF18_L37A	gaataaaacgGGCctcttgggcgtggaatg	*b
ph-mKGN-ORF18-R38A	S_8_KSHV_ORF18_R38A	caagagctgGCTtttattcatttggttctc	*b
	As_8_KSHV_ORF18_R38A	gaataaaAGCcagctcttgggcgtggaatg	*b
ph-mKGN-ORF18-H41A	S_9_KSHV_ORF18_H41A	gttttattGCAttggttctctgcaggatg	*b
	As_9_KSHV_ORF18_H41A	gagaaccaaTGCaataaaacgcagctcttg	*b
ph-mKGN-ORF18-L44A	S_10_KSHV_ORF18_L44A	catttggttGCCtgcaggatgtacaatttc	*b
	As_10_KSHV_ORF18_L44A	catcctgcaGGCaaccaaatgaataaaacg	*b
ph-mKGN-ORF18-P156A	S_11_KSHV_ORF18_P156A	gtgtgctcGCAtgcaggctgctccttgcg	*b
	As_11_KSHV_ORF18_P156A	cagcctgcaTGCgagcacacttcccagattaaac	*b
ph-mKGN-ORF18-R158A	S_12_KSHV_ORF18_R158A	cctgcGCTctgctccttgcggcgggc	*b
	As_12_KSHV_ORF18_R158A	cgcaaggagcagAGCgcaggggagcacacttcc	*b
ph-mKGN-ORF18-L159A	S_13_KSHV_ORF18_L159A	cctgcaggGCGctccttgcggcgggctac	*b
	As_13_KSHV_ORF18_L159A	cgcaaggagCGCcctgcaggggagcacacttc	*b
ph-mKGN-ORF18-C166A	S_14_KSHV_ORF18_C166A	ggctacGCActcgccttttggggcagc	*b
	As_14_KSHV_ORF18_C166A	caaaaggcgagTGCgtagcccgccgcaaggag	*b
ph-mKGN-ORF18-L167A	S_15_KSHV_ORF18_L167A	ggctactgcGCTgccttttggggcagcgatg	*b
	As_15_KSHV_ORF18_L167A	caaaaggcAGCgcagtagcccgccgcaag	*b
ph-mKGN-ORF18-W170A	S_16_KSHV_ORF18_W170A	cgcctttGCAggcagcgatgaacacgaac	*b
	As_16_KSHV_ORF18_W170A	catcgctgccTGCaaaggcgaggcagtagcc	*b
ph-mKGN-ORF18-G171A	S_17_KSHV_ORF18_G171A	cgccttttggGCCagcgatgaacacgaacg	*b
	As_17_KSHV_ORF18_G171A	catcgctGGCccaaaaggcgaggcagtag	*b

Plasmid name	Primer name	Primer sequences (5' -> 3')	
[ORF18 ASC mutants]			
ph-mKGN-ORF18_ASC1	S mm1 EcoRI KSHV ORF18	catcgaattcgatgctcGCAaaatacgtgtgtgagac	*c
ph-mKGN-ORF18_ASC2	S mm2 KSHV ORF18	CAGCGcttaggaggGCCatgtggcgctttttgca	*b
	As_mm2_KSHV_ORF18	ctcctaagCGCTGCgctcagcggctcggtctc	*b
ph-mKGN-ORF18_ASC3	S mm3 KSHV ORF18	GCAcaaaataagaatGCAaatacattccacgcccaag	*b
	As_mm3_KSHV_ORF18	TGCattcttattttgTGCaaagcgccacatgagcctc	*b
ph-mKGN-ORF18_ASC4	S_mm4_KSHV_ORF18	caaGCAGCCGCTtttattcatttggttctc	*b
	As_mm4_KSHV_ORF18	AGCGGCTGCttgggcgtggaatgtatttaaattc	*b
ph-mKGN-ORF18_ASC5	S_mm5_KSHV_ORF18	GCAttggttGCCtgcaggatgtacaatttc	*b
	As_mm5_KSHV_ORF18	GGCaaccaaTGCaataaaacgcagctcttgg	*b
ph-mKGN-ORF18_ASC6	S_mm6_KSHV_ORF18	GCTgtgGCCGCGttaagggaggctactgc	*b
	As_mm6_KSHV_ORF18	CGCGGCcacAGCtagaccgaaattgtacatc	*b
ph-mKGN-ORF18_ASC7	S_mm7_KSHV_ORF18	GCAgtgGCTGCAGCCtttttgtttaatctggg	*b
	As_mm7_KSHV_ORF18	CAGCcacTGCgttttccggggcgatgcg	*b
ph-mKGN-ORF18_ASC8	S_mm8_KSHV_ORF18	GCGGCAGCTctgggaagtgtgctcccc	*b
	As_mm8_KSHV_ORF18	gAGCTGCCGCaaagtttccgtccaccagg	*b
ph-mKGN-ORF18_ASC9	S_mm9_KSHV_ORF18	GCTGCAGCTgtgctcccctgcaggctg	*b
	As_mm9_KSHV_ORF18	gggagcacAGCTGCAGCattaaacaaaaagtttccg	*b
ph-mKGN-ORF18_ASC10	S_mm10_KSHV_ORF18	CAtgcGCTGCGctccttgcggcgggctac	*b
	As_mm10_KSHV_ORF18	CGCAGCgcaTGCgagcacacttcccagattaaac	*b
ph-mKGN-ORF18_ASC11	S_mm11_KSHV_ORF18	CAGCTgcctttGCAGCCagcgatgaacacgaacg	*b
	As_mm11_KSHV_ORF18	CaaaggcAGCTGCgtagcccgccgcaaggag	*b
ph-mKGN-ORF18_ASC12	S_mm12_KSHV_ORF18	CAcgctggGCTGCCttcttcgcccagaagcttttc	*b
	As_mm12_KSHV_ORF18	GCccagcgTGCgtgttcatcgctgccccaaaag	*b
ph-mKGN-ORF18_ASC13	S_mm13_KSHV_ORF18	GCAatttgctacGCGatagtctccgggcgtcttatg	*b
	As_mm13_KSHV_ORF18	CGCgtagcaaatTGCaagcttctgggcgaagaag	*b
ph-mKGN-ORF18_ASC14	S_mm14_KSHV_ORF18	ccGCAcgtGCTatgccacagaggtctctg	*b
	As_mm14_KSHV_ORF18	AGCacgTGCggagactatcaggtagcaaatg	*b
[ORF18 truncated mutants]			
ph-mKGN-ORF18Δ1	S_EcoRI_d1_KSHV-ORF18	gccacGAATTCgtggcgctttttgcaaaataagaatttaaatac	*a
ph-mKGN-ORF18Δ2	S_d2_KSHV-ORF18-2	GAGGCTCATGcatttggttctctgcaggatg	*d
	As_d2_KSHV-ORF18-2	gaaccaaatgCATGAGCCTCCTAAGCCCC	*d
ph-mKGN-ORF18Δ3	S_d3_KSHV-ORF18	GCGTTTTATCactgccaatgccgggacctac	*d
	As_d3_KSHV-ORF18	cattggcagtGATAAAACGCAGCTCTTGGGC	*d
ph-mKGN-ORF18Δ4	S_d4_KSHV-ORF18	GTTAAGGGAGGCTtggaagctcgtgtacgatgg	*d
	As_d4_KSHV-ORF18	cgagcttccaAGCCTCCCTTAACAGGTACAC	*d
ph-mKGN-ORF18Δ5	S_d5_KSHV-ORF18	AGGTGgaggcataccgggacagcc	*d
	As_d5_KSHV-ORF18	ggtatgcctcCACCTCCGCAGGAACCTTG	*d
ph-mKGN-ORF18Δ6	S_d6_KSHV-ORF18	GCTGCTGTGTctggcgaattatctgtttcacc	*d
	As_d6_KSHV-ORF18	gataattcgccagACACAGCAGCATCTCGCTG	*d
ph-mKGN-ORF18Δ7	S_d7_KSHV-ORF18	CTTGAGGGGCaacctggtggacggaaactttttg	*d
	As_d7_KSHV-ORF18	ccaggttGCCCCTCAAGAGCCCCAC	*d
ph-mKGN-ORF18Δ8	S_d8_KSHV-ORF18-2	CCCCGGAActtgcggcgggctactg	*d
	As_d8_KSHV-ORF18-2	ccgcaagTTCCGGGGCGATGCGAAC	*d
ph-mKGN-ORF18Δ9	S_d9_KSHV-ORF18	CAGGCTGCTCttcttcgcccagaagcttttc	*d
	As_d9_KSHV-ORF18	gcgaagaaGAGCAGCCTGCAGGGGAG	*d
ph-mKGN-ORF18Δ10	S_d10_KSHV-ORF18	CTGGGTGCGTaggtctctgctagtttgggc	*d
	As_d10_KSHV-ORF18	cagagacctACGCACCCAGCGTTCGTG	*d
ph-mKGN-ORF18Δ11	S_d11_KSHV-ORF18	CTTATGCCACAGcgcgacatccgctccatgtac	*d
	As_d11_KSHV-ORF18	gatgtcgcgCTGTGGCATAAGACGCCC	*d
ph-mKGN-ORF18Δ12	S_d12_KSHV-ORF18	GCAGTCTGTccgtccgaagcgcagc	*d
	As_d12_KSHV-ORF18	cttcggacggACAGACTGCCTCCACCG	*d
ph-mKGN-ORF18Δ13	As(stp)_ NotI _d13_KSHV-ORF18	taaGCGGCCGCttaagccggaagataacccg	*a
[Sub-cloning into 2xS tag expression plasmids]			
pCIneo-2xS-ORF18 WT/mut	S_EcoRI_ORF18	catGAATTCatgctcggaaaatacgtgtgtg	*a
	As_ORF18(stp)_MluI	taaACGCGTttaaaccgcgttgttgttaaac	*a

**a* : Uppercase indicates restriction enzyme site

**b* : Uppercase indicates mutagenesis sites

**c* : Uppercase indicates mutagenesis sites, underlined lowercase indicates restriction enzyme site

**d* : Uppercase indicates N-terminal protein coding sequences, lowercase indicates C-terminal protein coding sequences
